# MITOsym®: A Mechanistic, Mathematical Model of Hepatocellular Respiration and Bioenergetics

**DOI:** 10.1007/s11095-014-1591-0

**Published:** 2014-12-12

**Authors:** Y. Yang, S. Nadanaciva, Y. Will, J. L. Woodhead, B. A. Howell, P. B. Watkins, S. Q. Siler

**Affiliations:** 1 Institute for Drug Safety Sciences, The Hamner Institutes for Health Sciences, Research Triangle Park, North Carolina USA; 2 Compound Safety Prediction, Worldwide Medicinal Chemistry, Pfizer Inc, Groton, Connecticut 06340 USA

**Keywords:** bioenergetics, extracellular acidification rate, mechanism-based, mitochondrial toxicity, oxygen consumption rate

## Abstract

**Purpose:**

MITOsym, a new mathematical model of hepatocellular respiration and bioenergetics, has been developed in partnership with the DILIsym® model with the purpose of translating *in vitro* compound screening data into predictions of drug induced liver injury (DILI) risk for patients. The combined efforts of these two models should increase the efficiency of evaluating compounds in drug development in addition to enhancing patient care.

**Methods:**

MITOsym includes the basic, essential biochemical pathways associated with hepatocellular respiration and bioenergetics, including mitochondrial oxidative phosphorylation, electron transport chain activity, mitochondrial membrane potential, and glycolysis; also included are dynamic feedback signals based on perturbation of these pathways. The quantitative relationships included in MITOsym are based primarily on published data; additional new experiments were also performed in HepG2 cells to determine the effects on oxygen consumption rate as media glucose concentrations or oligomycin concentrations were varied. The effects of varying concentrations of FCCP on the mitochondrial proton gradient were also measured in HepG2 cells.

**Results:**

MITOsym simulates and recapitulates the reported dynamic changes to hepatocellular oxygen consumption rates, extracellular acidification rates, the mitochondrial proton gradient, and ATP concentrations in the presence of classic mitochondrial toxins such as rotenone, FCCP, and oligomycin.

**Conclusions:**

MITOsym can be used to simulate hepatocellular respiration and bioenergetics and provide mechanistic hypotheses to facilitate the translation of *in vitro* data collection to predictions of *in vivo* human hepatotoxicity risk for novel compounds.

**Electronic supplementary material:**

The online version of this article (doi:10.1007/s11095-014-1591-0) contains supplementary material, which is available to authorized users.

## Introduction

Drug-induced liver injury (DILI) is a significant issue in drug development, both preclinically and clinically. While the incidence of human DILI is relatively low (1), drugs associated with DILI lead to substantial cost for drug developers due to mostly late stage attrition. As such, the pharmaceutical industry and regulatory agencies are devoting substantial resources toward improving the preclinical and clinical screening processes. Liver injury is known to be multifactorial with mitochondrial toxicity being one of the known contributors. Recent advances in mitochondrial function assays have enhanced the ability to screen compounds for possible mitochondrial toxicity liabilities (2–5). These include high-throughput cellular respiration measurement systems such as the Seahorse Biosciences XF analyzers (6). The XF analyzer provides real time measurement of cellular bioenergetics in whole cells. These new assay systems provide an advantage over the classic isolated mitochondrial respiration assays by providing an environment that more closely replicates the native, *in vivo* milieu (4). These cell-based assay systems also provide the ability to estimate oxygen consumption, glycolysis flux and adaptive, compensatory changes during periods of mitochondria duress (7).

Mechanistic mathematical models are available to assist in evaluating compounds for hepatotoxicity liabilities. One such example is the DILIsym® model, which provides the ability to predict DILI based on preclinical *in vitro* and/or *in vivo* data (8). Mathematical models can assist in analyzing and interpreting complex data sets, understanding mechanisms of drug action, generating testable hypotheses, and guiding experimentation. The information provided by the simulations can help minimize experimentation, thereby saving time and effort. Moreover, simulations of ‘what if’ scenarios can provide researchers with the ability to predict experimental outcomes and make decisions that can improve patient safety.

**Fig. 1:**
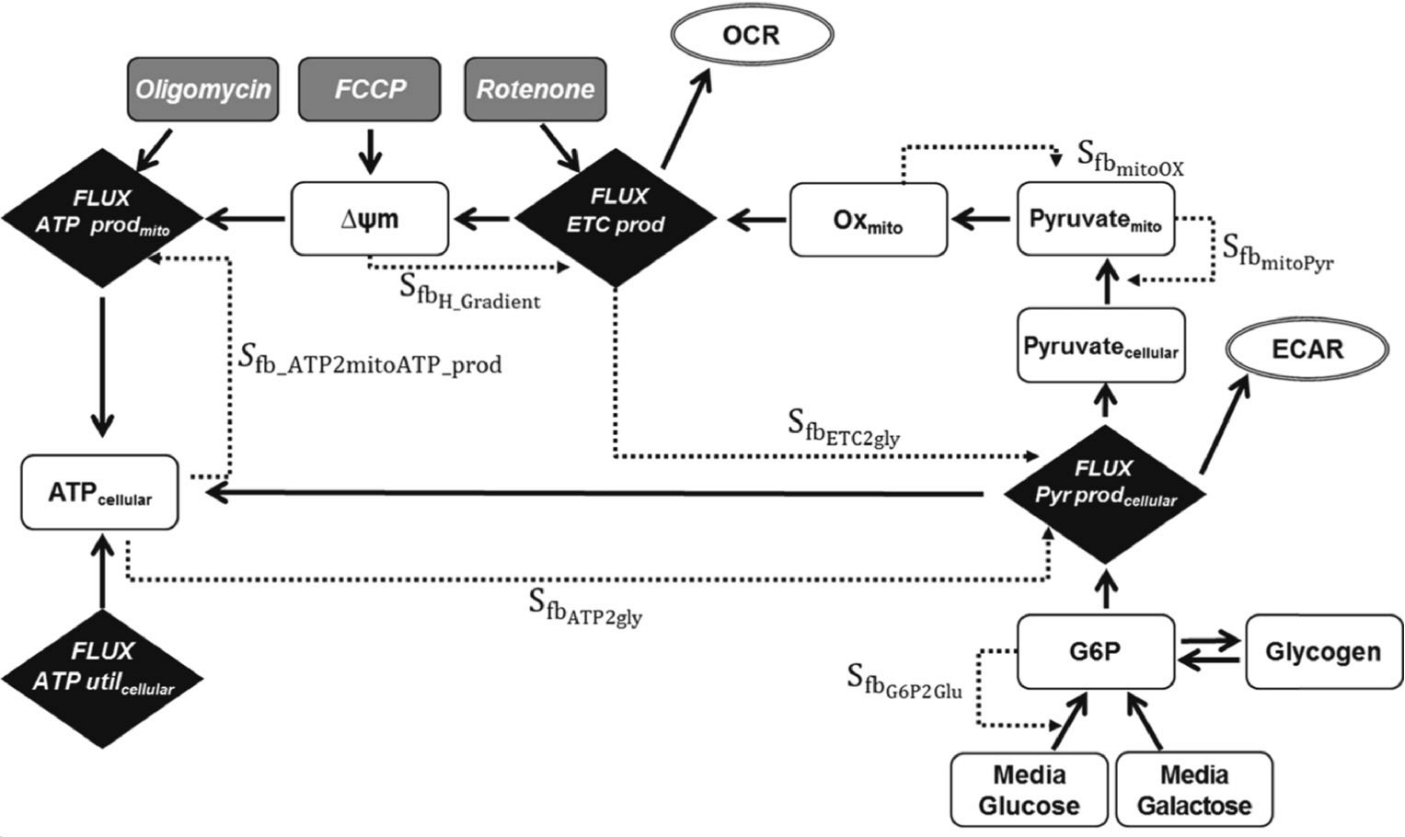
Schematic diagram of MITOsym: MITOsym includes key biochemical processes for cellular and mitochondrial bioenergetic responses. Classic effectors (oligomycin, FCCP and rotenone) were used to investigate mechanism-specific mitochondrial toxicity. Cellular feedback signals were used to maintain mitochondrial homeostasis or modulate mitochondrial response to external stressors. Primary model outputs include oxygen consumption rate (OCR), extracellular acidification rate (ECAR) and ATP. State variables in MITOsym are displayed as white rectangles, while fluxes are shown in black diamonds. Feedback signals are denoted by dashed lines. Descriptions of the equations describing the state variables, fluxes, and feedback signals are given in Tables II, III and IV and Appendix B.

This paper describes a new mechanistic, mathematical model of hepatocellular respiration and bioenergetics, called MITOsym. MITOsym simulates and predicts bioenergetics in the presence or absence of compounds that modulate mitochondrial function in HepG2 cells. The model contains many essential bioenergetic components such as mitochondrial oxidative phosphorylation, electron transport chain activity, mitochondrial membrane potential, glycolysis, and cellular pathways for metabolic regulation of bioenergetics as illustrated in Fig. 1. The effector compounds have been chosen for their well-characterized effects on different aspects of mitochondria function; rotenone is an inhibitor of complex I of the electron transport chain, oligomycin is an inhibitor of the F_1_F_0_ ATPase, and FCCP uncouples the mitochondria proton gradient. Characterizing the disruption of each of these processes with these compounds has elucidated quantitation of key feedback signals within and beyond mitochondria. The model has been designed and calibrated with a variety of data, much of it collected using the Seahorse Biosciences XF analyzer. While considerable amounts of the data that were used to construct the model were publically available in the literature, we also conducted additional experiments to generate specific sets of data; these data are described in this report as well.

MITOsym has also been designed to provide inputs into DILIsym® and facilitate the prediction of *in vivo* hepatotoxicity based on *in vitro* compound data. DILIsym® and MITOsym software are developed under the DILI-sim Initiative, a pre-competitive partnership between the Hamner-UNC Institute for Drug Safety Sciences and 14 pharmaceutical companies. For more information on access to MITOsym and DILIsym®, see www.dilisym.com. This simulation partnership should increase the efficiency and effectiveness of evaluating the potential for new compounds with mitochondria liabilities to elicit DILI.

## Materials and Methods

### Materials

All chemicals were purchased from Sigma-Aldrich (St. Louis, MO) and were of the highest purity available. The fluorescence dyes, tetramethylrhodamine methyl ester (TMRM) and Hoechst 33342, were from Invitrogen (Carlsbad, CA). Cell culture media and supplements were from Invitrogen (Carlsbad, CA). Low-buffered RPMI medium was from Molecular Devices (Sunnyvale, CA). XF96 sensor cartridges and XF96-well plates were from Seahorse Bioscience (Billerica, MA).

### Cell Culture Conditions for HepG2 Cells

HepG2 cells were obtained from the American Type Culture Collection (Manassas, VA). Cells were grown in Dulbecco’s modified Eagle’s medium (DMEM) (Invitrogen 11885–084) containing 5.5 mM glucose and 1 mM sodium pyruvate and supplemented with 10% fetal bovine serum, 5 mM 4-(2-hydroxyethyl) piperazine-1-ethanesulfonic acid (HEPES), and 100 units/mL penicillin-streptomycin in a 37°C, 5% CO_2_ humidified atmosphere.

### Experimental Protocol 1: Measurement of the Effect of FCCP on the Mitochondrial Membrane Potential of HepG2 Cells

Mitochondrial membrane potential was measured based on the accumulation of TMRM. HepG2 cells were seeded in 96-well plates at 10,000 cells/well in culture medium and incubated overnight in a humidified 37°C, 5% CO_2_ atmosphere. The following day, cells were incubated with carbonyl cyanide 4-(trifluoromethoxy) phenylhydrazone (FCCP) at various concentrations in culture medium for 1 h at 37°C, 5% CO_2_. TMRM and Hoechst 33342 were then added to each well (at a final concentration of 100 nM and 4 μg/ml, respectively) and the cells incubated at 37°C, 5% CO_2_ for 30 min. The cells were rinsed three times with Hanks Balanced Salt Solution (HBSS; 200 μl/well); the final HBSS rinse was not aspirated.

Automated live-cell image acquisition was performed on a Thermo Fisher Scientific Cellomics® ArrayScan® VTI High Content Screening Reader using a 10X objective. An XF93-Hoechst filter and an XF93-TRITC filter were used to image Hoechst 33342 and TMRM, respectively. Image acquisition was set to a minimum of 500 objects per well. Image analysis was done using the Compartmental Analysis Bioapplication (Thermo Fisher Scientific). Mitochondrial membrane potential staining due to TMRM was defined as the punctate signal around the Hoechst-stained nucleii. Raw data were exported from vHCSView (Thermo Fisher Scientific), and IC_50_ values were generated using GraphPad Prism 5 (San Diego, CA) with non-linear regression analysis.

### Experimental Protocol 2: Measurement of the Oxygen Consumption Rate and Extracellular Acidification Rate of HepG2 Cells in Media Containing Different Glucose Concentrations

Oxygen consumption rates (OCR) and the extracellular acidification rates (ECAR) of HepG2 cells were measured in real-time, simultaneously, in an XF96 Extracellular Flux Analyzer (Seahorse Bioscience, Billerica, MA) essentially as described in Nadanaciva *et al.* (3) with some modifications. Cells were seeded in XF96-well plates at 24,000 cells/80 μL culture medium/well and incubated in a 37°C, 5% CO_2_ humidified atmosphere for 24 h.

To measure the effect of various concentrations of glucose on the OCR, ECAR, and respiratory capacity of HepG2 cells, the culture medium from the XF96 cell plates was aspirated the day after seeding the cells, and the cells rinsed three times in pre-warmed assay buffer (111 mM NaCl, 4.7 mM KCl, 2 mM MgSO_4_, 1.2 mM Na_2_HPO_4_), pH 7.4. The cells were then maintained in 150 μL/well assay buffer supplemented with various concentrations of glucose at 37°C in a non-CO_2_ incubator for 60 min.

This cellular environment, including media glucose of 5.5 mM but excluding any mitochondrial effector drugs, is used as the basal condition in all MITOsym simulations.

To measure the reserve capacity of the cells, FCCP was diluted to 21 μM in assay buffer, and 25 μL pre-loaded into reagent delivery port A of each well in the XF96 sensor cartridge. After a 25 min calibration of the XF96 sensor cartridge, four measurements of the OCR and ECAR of the cells were made using a 2 min mix, 5 min measure cycle. FCCP was then injected and six OCR and ECAR measurements were made (the final concentration of FCCP in each well was 3 μM). The OCR and ECAR immediately prior to FCCP injection were used as the basal OCR and ECAR, respectively. The first OCR and ECAR obtained after FCCP injection were used to determine the reserve capacity, *i.e.* the percentage change in OCR and ECAR caused by FCCP.

### Experimental Protocol 3: The effect of Oligomycin on OCR and ECAR

To examine the effect of oligomycin on the OCR and ECAR of HepG2 cells, cells were seeded in XF96-well plates and the culture medium was aspirated after 24 h. The cells were rinsed three times in pre-warmed serum-free low-buffered RPMI medium, pH 7.4. The cells were then maintained in 150 μL/well of serum-free low-buffered RPMI medium at 37°C in a non-CO_2_ incubator for 60 min. A compound plate containing oligomycin at various concentrations in serum-free low-buffered RPMI medium supplemented with 1% (v/v) DMSO was prepared. OCR and ECAR measurements were collected before and after addition of oligomycin as described in Experimental protocol 2 (above). The OCR and ECAR immediately prior to the oligomycin/vehicle injection were used as the basal OCR and ECAR, respectively, and were defined as 100% when generating the oligomycin concentration response curves. The first OCR and ECAR obtained after oligomycin injection were used to determine the percentage change in OCR and ECAR caused by the compound.

#### Additional Data from Published Literature

Data from Nadanaciva *et al.* (3) were used to examine the effects of increasing concentrations of rotenone and FCCP on OCR and ECAR of HepG2 cells, as measured with an XF96 Analyzer. Data from more complex protocols were also included from other publications (9–11); OCR was measured using an XF24 analyzer during and after the serial administration of 1 uM oligomycin, 1 uM FCCP, and 1 uM rotenone to HepG2 cells. These data were used to support the modeling efforts by providing quantitative information about dynamic, cellular adaptation.

Data from a study by Marroquin *et al.* (2) were also included to help characterize the relative contributions from mitochondria and glycolysis to total cellular ATP production. The study included measurement of ATP at varying concentrations of oligomycin, FCCP, and rotenone in HepG2 cells cultured with 25 mM glucose or 10 mM galactose as the primary oxidative substrate in the media. Cells cultured in media containing galactose are unable to generate net ATP from glycolysis and thus rely on their mitochondria for ATP productions. Cellular ATP content was measured after 24 h of incubation with each compound. These data were used to support the modeling efforts by providing quantitative information about the dynamic interplay between mitochondria and glycolytic ATP production. Table I summarizes the time-series and dose–response data used to optimize the changes in cellular bioenergetics and/or respiration (relative to basal) in response to the culture conditions and exemplar compounds (rotenone, FCCP and oligomycin) for HepG2 cells.

**Table I:** Summary of the Experimental Data Used to Describe the Bioenergetic Responses to Various Mitochondrial Effectors in HepG2 Cell

	Glucose	FCCP	Rotenone	Oligomycin	Multiple drug
OCR	Measured	Nadanaciva *et al.* (2012)	Nadanaciva *et al.* (2012)	Measured	Felser *et al.* (2013), Zahno *et al.* (2011) and Mullen *et al.* (2011)
Gradient		Measured			
ECAR	Measured	Nadanaciva *et al.* (2012)	Nadanaciva *et al.* (2012)	Measured	
ATP		Marroquin *et al.* (2007)	Marroquin *et al.* (2007)	Marroquin *et al.* (2007)	

#### Computational Modeling of Bioenergetics

##### Metabolic Description

A mechanism-based, computational model, MITOsym, was developed to describe hepatocellular bioenergetics in hepatocytes in basal conditions and when exposed to compounds that modulate mitochondrial function. MITOsym is seated in the MATLAB computing platform (The MathWorks, Natick, MA) and is composed of ordinary differential equations, algebraic expressions, and a corresponding graphical user interface. MITOsym includes mathematical descriptions of many of the essential biochemical components of bioenergetics, as illustrated in Fig. 1. Two energy-generating pathways, glycolysis and mitochondrial oxidative phosphorylation (OXPHOS) were included. Glycolysis supports cellular bioenergetics in MITOsym by directly supplying extra-mitochondrial ATP in addition to providing substrates for mitochondrial electron transport chain activity (ETC) activity and ATP production (12). For mitochondrial OXPHOS, similar to the approaches described in Hafner *et al.* (13) and Ainscow *et al.* (14), the membrane potential was used as an intermediate control point which was supported by ETC activity and dispatched *via* ATP synthesis and uncoupling. This design is in agreement with observations of decreased proton gradient and ATP levels in isolated mitochondria or hepatocytes treated with complex I/II inhibitors (15,16) or uncouplers (15).

We have simplified some aspects of cellular and mitochondrial biochemistry in an effort to reduce the complexity and number of equations in MITOsym. The focus of our model is to capture key aspects of mitochondria within the whole cell context. This allows us to incorporate key intracellular feedback signals that have direct and indirect effects on mitochondrial function (*e.g.*, increased glycolysis in response to compromised mitochondrial function). Within this cellular context, however, certain aspects of mitochondrial biochemistry are not experimentally measured such as mitochondria pH and mitochondrial ion transport. While other models have focused on isolated mitochondria and include these specific components, the cellular focus of MITOsym has precluded explicitly including them.

NADH and the components of the tricarboxylic acid cycle (aka Krebs cycle) have not been explicitly included into MITOsym. In most cell culture conditions, the media provides ample substrate to maintain the levels of TCA intermediates and support ETC flux; anapleurosis is not limiting. Moreover, compounds with hepatotoxicity liabilities have by and large not included TCA cycle inhibition as a mechanism. The production and utilization of NADH is not explicitly represented in MITOsym, but its dynamics are somewhat represented by the utilization of pyruvate. We have included a mitochondrial pyruvate state variable upon which ETC flux is dependent. While mitochondrial pyruvate levels are quite low, this state variable also represents the dynamics of mitochondrial NADH turnover. Thus specific disruptions in NAHD/NAD ratios (*i.e.*, redox state) can be represented by introducing changes in pyruvate oxidation in MITOsym.

The details of each section of MITOsym are described below.

- **Glycolysis**
- Glycolysis is an anaerobic pathway that converts glucose into pyruvate and generates ATP in the cytosol (17). The glycolysis pathway is primarily represented by the dynamic changes in the intermediates, glucose-6 phosphate and pyruvate, as illustrated in Fig. 1 and as described in more detail as follows. Glucose uptake and glucose-6-phosphate (G6P) production:

Components comprising glucose uptake, and glucose-6-phosphate (G6P) production were modeled using differential equations (see, Table II, s1 through s5).

**Table II:** Metabolic Reactions and the Basal Steady State Values Computed for State Variables. See Appendix B for More MITOsym®equation Descriptions

	State variable	Differential equation for hepatica bioenergetics reactions	Basal SS value for glucose media	Basal SS value for galactose media
s1	[drug_cellular_]	$\frac{d{\left[ drug\right]}_{cellular}}{dt} = Flu{x}_{uptake}^{drug}- Flu{x}_{cellular}^{drug}$	0 [mM]	0 [mM]
s2	[G6P]	$\frac{d\left[G6P\right]}{dt}= Eff\_ Flu{x}_{prod}^{G6P}- Eff\_ Flu{x}_{prod,\ cellular}^{pyr}*0.5- Flu{x}_{prod}^{gly}+ Flu{x}_{degrad}^{gly}$	1 [mM]	1 [mM]
s3	[Glycogen]	$\frac{d\left[ Glycogen\right]}{dt}= Flu{x}_{prod}^{gly}- Flu{x}_{degrad}^{gly}$	0.001 [mM]	0 [mM]
s4	[Pyruvate_cellular_]	$\frac{d\left[Py{r}_{cellular}\right]}{dt}= Eff\_ Flu{x}_{prod,\ cellular}^{pyr}- Eff\_ Flu{x}_{prod,\ mito}^{pyr}- Flu{x}_{prod}^{lac}$	5 [mM]	4.7 [mM]
s5	[Pyruvate_mito_]	$\frac{d{\left[Pyr\right]}_{mito}}{dt} = Eff\_ Flu{x}_{prod,\ mito}^{pyr}- Eff\_ Flu{x}_{prod}^{pyr2 OX}$	5 [mM]	4.7 [mM]
s6	[OX_mito_]	$\frac{d{\left[ OX\right]}_{mito}}{dt} = Eff\_ Flu{x}_{prod}^{pyr2 OX}- Eff\_ Flu{x}_{prod}^{ETC}$	0.3 [mM]	0.4 [mM]
s7	*ΔΨ*_*m*_	$\frac{d{\varPsi}_m}{dt}=Conversio{n}_f\times Eff\_Flu{x}_{prod}^{ETC}-Eff\_Flu{x}_{prod, mito}^{ATP}-Uncouplin{g}_{drug}$	172.2 [mM]	169.4 [mM]
s8	[ATP_cellular_]	$\frac{d{\left[ATP\right]}_{cellular}}{dt} = Eff\_ Flu{x}_{mito,\ lowgrad}^{ATP}+ Eff\_ Flu{x}_{gly}^{ATP}-k*{\left[ATP\right]}_{cellular}$	4 [mM]	4 [mM]

*In vitro*, hepatocytes take up glucose from culture media and subject it to phosphorylation, generating glucose 6-phosphate (G6P). G6P has numerous possible fates, including being catabolized into pyruvate *via* glycolysis, or stored as glycogen. Each of these components and processes is described in the mathematical equations of the model for simulating mitochondrial function. It is assumed that the glucose concentration in the hepatocyte was in equilibrium with the media glucose concentration. Moreover, the media glucose concentration was assumed to be constant throughout each experiment and simulation. Glucose uptake and phosphorylation was modeled as a saturable process. The metabolic flux of G6P (*Flux*_*prod*_^*G*6*P*^) is estimated under steady-state conditions from flux balance analysis (Appendix A; Table III). The Km (Michaelis-Menten constant) of G6P production is estimated by manually adjusting the parameter value to fit the basal and maximum respiratory dose–response data collected for various glucose concentrations. The estimated value of *Flux*_*prod*_^*G*6*P*^ is 1.875 mM/min where synthesis of G6P is a saturable response with Km in HepG2 cells set to 10 mM, based on Km of glucokinase reported *in vivo* (18). In the model, the basal hepatocyte G6P concentration was set to 1 mM (18).

**Table III:** Metabolic Fluxes at Steady State Given Different Cell Culture Media Conditions. See Appendix B for More MITOsym®equation Descriptions

		Description	Glucose	Galactose
F1	*Flux*_*prod*_^*G*6*P*^	Rate of hepatocyte glucose/G6P uptake and phosphorylation, described as a Michaelis-Menten reaction	1.875 [mM/min]	2.13[mM/min]
F2	*Flux*_*prod*, *cellular*_^*pyr*^	Rate of hepatocyte total pyruvate production *via* glycolysis, described as a Michaelis-Menten reaction	3.75 [mM/min]	4.26[mM/min]
F3	*Flux*_*prod*_^*lac*^	Rate of lactate production from pyruvate, described as a first-order reaction	3.15 [mM/min]	3.45[mM/min]
F4	*Flux*_*prod*, *mito*_^*pyr*^	Rate of pyruvate use by mitochondria in absence of effectors is linear to total pyruvate production, *Flux*_*prod*, *cellular*_^*pyr*^	0. 6 [mM/min]	0.84[mM/min]
F5	*Flux*_*prod*_^*pyr*2*OX*^	Rate of pyruvate use as oxidative substrate in mitochondria, described as a Michaelis-Menten reaction	0. 6 [mM/min]	0.84[mM/min]
F6	*Flux*_*prod*_^*ETC*^	Rate of oxidative substrate used for ETC activity in mitochondria in absence of effectors is the sum of the oxidative substrate from mitochondrial pyruvate or fatty acid (the latter is not included in the current model)	0. 6 [mM/min]	0.84[mM/min]
F7	*Flux*_*prod*,*mito*_^*ATP*^	Rate of mitochondria ATP production in absence of effectors, described as a Michaelis-Menten reaction	1000 [pmol ATP/min]	1334.7 [pmol ATP/min]

Glycogen also participates in G6P flux *in vivo*, providing G6P during fasting periods and storing G6P in the fed state. *In vitro*, however, there is very little glycogen in HepG2 cells (19). Therefore, the glycogen concentration is set to 0.001 mM and the net G6P production from glycogen breakdown is zero in the model.

b) Pyruvate production: Pyruvate is primarily generated from G6P when HepG2 cells are cultured in media excluding lactate. The breakdown of each G6P molecule generates two pyruvate molecules in addition to two (net) ATP molecules. The estimated basal value of *Flux*_*prod*, *cellular*_^*pyr*^ is 3.75 mM/min where the generation of hepatic pyruvate from G6P is assumed to be near maximal with Km set to 0.01 mM and the cytosolic pyruvate concentration is set to 5 mM.
c) ECAR computation: Lactate is a primary product of pyruvate metabolism. As such, it is representative of glycolysis rates; extracellular acidification rates (ECAR) can be used as an indicator of lactate release and glycolysis (6). In our model, the simulated pyruvate-to-lactate metabolic flux is used to compute the ECAR. The basal lactate flux (*Flux*_*prod*_^*lac*^) is estimated as 3.15 mM/min given the reported basal ECAR is 66.2 mpH/min (3), the conversion factor is 4.28 $\frac{{\mathrm{pmoleH}}^{+}}{\mathrm{mpH}}$ (20), and cell volume is 9e^−8^ L for 24,000 cell (21). The mathematical equations and data used to define the stoichiometric relationship among metabolic flux are detailed in Appendix A (Intermediate fluxes #1) and Table II.
2) **ATP Production (Cytosol and Mitochondria)**

Glycolysis supports cellular bioenergetics by directly supplying extra-mitochondrial ATP and providing substrates for mitochondrial oxidative phosphorylation (OXPHOS) and mitochondria ATP production. ATP production in mitochondria is based on oxidative phosphorylation, the coupling of the mitochondria proton gradient with phosphorylation of ADP to generate ATP. The components of ATP production are described as follows:

- Oxidative substrate: Cytosolic pyruvate is transported into mitochondria and then converted to acetyl-CoA by pyruvate dehydrogenase (17). Acetyl CoA undergoes further oxidation through the tricarboxylic acid (TCA) cycle to generate the ETC substrates, nicotinamide adenine dinucleotide in its reduced form (NADH) and flavine adenine dinucleotide in its reduced form (FADH_2_). The MITOsym model simplifies the network of enzymes involved in these processes into a two-step process, where cytosolic pyruvate is converted to mitochondrial pyruvate which subsequently supports ETC enzyme complex activity. In the model, mitochondrial pyruvate is a discrete state variable that provides an intermediate pool controlling flux through the electron transport chain. The basal concentration of mitochondrial pyruvate is assumed to be equivalent to cytosolic pyruvate (*e.g.*, for hepatocytes, 5 mM), and the basal flux of mitochondrial pyruvate oxidation is 0.6 mM/min (detail shown in Appendix A). The Michaelis-Menten constant (Km) is 1 mM for this process. Mitochondrial pyruvate also serves as a regulator for mitochondria uptake of pyruvate from cytosol in our model as described in the cellular regulation section (Table IV, eq. EF3).

**Table IV:** Algebraic Expressions of Effective Flux Used for Intermediates

		Algebraic expressions
EF1	*Eff* _ *Flux*_*prod*_^*G*6*P*^	$Flu{x}_{prod}^{G6P}*\ {\mathrm{S}}_{{\mathrm{fb}}_{\mathrm{G}6\mathrm{P}2\mathrm{G}\mathrm{l}\mathrm{u}}}$
EF2	*Eff* _ *Flux*_*prod*, *cellular*_^*pyr*^	*Flux*_*prod*, *cellular*_^pyr^* ${\mathrm{S}}_{{\mathrm{fb}}_{\mathrm{ATP}2\mathrm{p}\mathrm{y}\mathrm{r}}}*{\mathrm{S}}_{{\mathrm{fb}}_{\mathrm{ETC}2\mathrm{p}\mathrm{y}\mathrm{r}}}$
EF3	*Eff* _ *Flux*_*prod*, *mito*_^*pyr*^	$Flu{x}_{prod,\ mito}^{pyr}*\ {\mathrm{S}}_{{\mathrm{fb}}_{\mathrm{mitoPyr}}}$
EF4	*Eff* _ *Flux*_*prod*_^*pyr*2*OX*^	$Flu{x}_{prod}^{pyr2 OX}*\ {\mathrm{S}}_{{\mathrm{fb}}_{\mathrm{mitoOX}}}$* ${\mathrm{S}}_{{\mathrm{fb}}_{\mathrm{H}\_\mathrm{Gradient}}}$
EF5	*Eff* _ *Flux*_*prod*_^*ETC*^	*Flux*_*prod*_^*ETC*^ * Drug_ETC _ inhibitor_
EF6	*Eff* _ *Flux*_*prod*,*mito*_^*ATP*^	$Flu{x}_{prod, mito}^{ATP}*{\mathrm{S}}_{{\mathrm{fb}}_{\mathrm{ATP}2\mathrm{mitoATP}\_\mathrm{prod}}}*{\mathrm{Drug}}_{\mathrm{ATP}\mathrm{ase}\_\mathrm{inhibitor}}$
EF7	*Eff* _ *Flux*_*mito*,*lowgradient*_^*ATP*^	$Eff\_ Flu{x}_{prod, mito}^{ATP}*\left(1-\mathrm{k}\cdot {\mathrm{S}}_{{\mathrm{fb}}_{\mathrm{MMP}2\mathrm{A}\mathrm{T}\mathrm{P}}}\right)$
EF8	*Eff* _ *Flux*_*gly*_^*ATP*^	$Eff\_ Flu{x}_{prod,\ cellular}^{pyr}*{\mathrm{S}}_{{\mathrm{Eff}}_{\mathrm{glycolysis}}}$
- Pyruvate oxidation. Pyruvate is primarily generated from G6P when HepG2 cells are cultured in media lacking lactate. The breakdown of each G6P molecule generates two pyruvate molecules in addition to two (net) ATP molecules. Pyruvate has two primary fates. It undergoes conversion to lactate in rates proportional to the amount of pyruvate. Mitochondrial utilization of pyruvate also occurs *via* the actions of pyruvate dehydrogenase, the tricarboxylic acid cycle enzymes, and the OXPHOS pathway. In the basal condition for the HepG2 cells, it was estimated that 16% of pyruvate flux (*Flux*_*prod*, *cellular*_^*pyr*^) was used for mitochondrial OXPHOS. This ratio is derived from the estimated ATP contributed from OXPHOS and glycolysis as described in the ATP production section, below, and in Appendix A (statement 7).
- ETC activity and respiration: Oxygen consumption rates (OCR) are used as an indicator of ETC activity (4), as oxygen is consumed in the final step of the electron transport chain. OCR in the MITOsym model is linearly proportional to ETC activity, (*Flux*_*prod*_^*ETC*^), which is based on the oxidation rate of mitochondrial pyruvate (*Flux*_*prod*_^*pyr*2*OX*^). The governing equations are given in Table II, equation s6. In the basal state, ETC activity and OCR are at 0.6 mmol/L/min and 200 pmol O_2_/min, respectively. The reported OCR value is 199.6 pmoleO_2_/min/24000 cell (3).
- Proton gradient: Mitochondrial ATP synthesis is dependent upon a gradient of protons (ΔΨm) that is established as protons are pumped out of the inner matrix into the inter-membrane space by the ETC (4,14). The MITOsym model includes a representation of ΔΨm (Table II, s7), computed as a state variable. The basal steady-state ΔΨm is maintained by two opposing processes: ETC activity leads to production of ΔΨm and ATP synthesis dissipates it. The basal value of ΔΨm is 172 mV in MITOsym, which agrees well with the physiological range of 180 ± 40 mV for isolated mitochondria (22).
- Mitochondrial ATP production: Mitochondria utilize ΔΨm to synthesize ATP *via* the F_1_F_O_ -ATPase. The rate of ATP synthesis in MITOsym is proportional to ΔΨm. In the basal state, the mitochondrial ATP production rate (Flux_prod,mito_^ATP^) is 1000 pmol/min (Appendix A) and the cellular ATP concentration is 4 mM, comparable to reported *in vitro* data generated for this cell type (18). Reductions in ΔΨm (*e.g.*, *via* uncoupling) lead to reductions in mitochondrial ATP production. Also, drugs or their metabolites directly inhibiting ATP synthase block H+ gradient dissipation. This leads to an increase in the simulated ΔΨm, which through a feedback manner reduces ETC flux in MITOsym.
- Total hepatocellular ATP production: Total hepatocellular ATP production includes ATP produced from mitochondria (OXPHOS) and the cytosol (glycolysis). Mitochondrial contributions to total cellular ATP production for HepG2 cells are approximately 75%, with the remainder generated by glycolysis when cultured in 5.5 mM glucose media . Using the basal OCR and ECAR values for the specific cell type of interest, in this case of 199.6 pmol O2/min and 66.2 mpH/min, the fractional contribution of ATP from OXPHOS and glycolysis was estimated to be 1000 and 337.2 pmol ATP/min respectively for HepG2 cells in MITOsym. Moreover, we estimated that 16% of glycolysis flux was used for mitochondrial pyruvate oxidation and 84% was released as lactate (Appendix A). This estimate was based on the presumed glucose yield of 36 ATP during mitochondrial oxidation and 2 ATP during glycolysis (See Appendix A for more details). Baseline hepatocyte ATP levels in MITOsym were based on ATP levels measured in human hepatocytes, and they were consistent with the reported ATP levels in HepG2 cells.

Basal ATP turnover (production and utilization) in MITOsym was designed to include total hepatocellular ATP production (including mitochondria and cytosol) and first order utilization. The steady state ATP turnover rate was based on human caloric expenditure data, and includes measured whole body basal metabolic rate, the fraction of the basal metabolic rate from the liver (or organ in which the cells of interest reside), the mass of the liver (or organ in which the cells of interest reside), and the weighted average of the energetic cost of synthesizing ATP from fatty acids, carbohydrates, and amino acids (as measured, or from published data). Although rare, the F_1_F_O_ ATPase can reverse the direction of the typical chemical reaction and hydrolyze ATP. In these situations, the mitochondria can contribute substantially to ATP utilization. MITOsym includes equations to account for net mitochondrial ATP production rates in this situation, and the predicted ATP level can be computed both including and excluding contributions from mitochondrial ATP hydrolysis (see also Table IV, equation EF6-7. The key algebraic expressions and ODE functions are detailed in Appendix B.

3) **Cellular regulation**

Cells often use intracellular feedback and metabolic regulation to maintain bioenergetic homeostasis. To include the regulation of metabolic pathways in the model in response to external stressors, pathway-specific signals were described mathematically as a function of the relevant state variables or intermediate products, and they were used to adjust metabolic fluxes as shown in Table IV. The diagram of the regulation network including the feedback signals is shown in Fig. 1, with descriptions of each signal given below.

- *Regulation between* ΔΨm and ETC *activity*

A feedback signal from ΔΨm to ETC activity was included in MITOsym based on data describing OCR changes in hepatocytes treated with FCCP and oligomycin. As an uncoupler, FCCP decreases the proton gradient and increases OCR (23). Oligomycin, which inhibits the F_1_F_O_ -ATPase, increases the proton gradient and reduces OCR (22,24). The MITOsym regulation signal based on changes in ΔΨm (${\mathrm{S}}_{{\mathrm{fb}}_{\mathrm{H}\_\mathrm{Gradient}}}$) modulates mitochondrial pyruvate utilization (*Eff* _ *Flux*_*prod*_^*pyr*2*OX*^) as shown in Table III. ${\mathrm{S}}_{{\mathrm{fb}}_{\mathrm{H}\_\mathrm{Gradient}}}$ is described with a non-linear Hill equation (Eq. S1):

S1 $$ {\mathrm{S}}_{{\mathrm{fb}}_{\mathrm{H}\_\mathrm{Gradient}}}={{\mathrm{Km}}_{\mathrm{NegFeedA}}}^{\mathrm{n}}/\left({{\mathrm{Km}}_{\mathrm{NegFeedA}}}^{\mathrm{n}}+\varDelta \varPsi {\mathrm{m}}^{\mathrm{n}}\right) $$

A Hill coefficient (*n* = 30) and Km of 165.7 mV was used to generate a sigmoidal shape where a mild loss of gradient will result in a steep increase of ${\mathrm{S}}_{{\mathrm{fb}}_{\mathrm{H}\_\mathrm{Gradient}}}$. The magnitude of this signal was based on the FCCP data (3) where $\frac{\mathrm{Max}\left({\mathrm{S}}_{{\mathrm{fb}}_{\mathrm{H}\_\mathrm{Gradient}}}\right)}{\mathrm{basal}\left({\mathrm{S}}_{{\mathrm{fb}}_{\mathrm{H}\_\mathrm{Gradient}}}\right)}$ =4 to allow a comparable increase in ETC activity.

b) *Regulation between mitochondrial function and glycolysis*

Glycolysis can increase during periods of mitochondrial duress, as evidenced by increases in ECAR in the presence of various mitochondrial inhibitors and uncoupling agents (3,16). Cytosolic ATP production associated with the increased glycolysis helps compensate for decreases in mitochondrial ATP production. Intuitively, the state of ATP balance (total ATP) would be an ideal regulator of glycolysis. Cellular ATP/ADP ratios have been reported to regulate glycolytic flux (25), and changes in cellular ATP concentrations are used in MITOsym as a controller of glycolytic flux as well. A second regulatory link between mitochondrial function and glycolysis was included in MITOsym as well. ECAR increases in the presence of mitochondrial inhibitors have been observed in several reports (3,26), and a correlation has been established between the OCR reduction and the ECAR increase (27). As such, the relative change in ETC activity also participates in regulating glycolysis flux. The equations for these two regulatory signals (Eq. S2: relative change of OCR level $\left({\mathrm{S}}_{{\mathrm{fb}}_{\mathrm{ETC}2\mathrm{g}\mathrm{l}\mathrm{y}}}\right)$ and Eq. S3: cellular ATP concentration $\left({\mathrm{S}}_{{\mathrm{fb}}_{\mathrm{ATP}2\mathrm{g}\mathrm{l}\mathrm{y}}}\right)$ are as follows:

S2 $$ {S}_{f{b}_{ATP2gly}}=\left(1-\mathrm{K}\right)+\mathrm{K}\cdot \left(\frac{2\times \mathrm{Basal}\_{\mathrm{ATP}}_{\mathrm{total}}}{{\mathrm{Basal}}_{{\mathrm{ATP}}_{\mathrm{total}}}+\mathrm{Current}\_{\mathrm{ATP}}_{\mathrm{total}}}\right) $$

S3 $$ {\mathrm{S}}_{{\mathrm{fb}}_{\mathrm{ETC}2\mathrm{g}\mathrm{l}\mathrm{y}}}=1+\mathrm{K}\cdot \mathrm{abs}\left(\frac{Flu{x_{prod}^{ETC}}_{\mathrm{current}}}{Flu{x_{prod}^{ETC}}_{\mathrm{basal}}}-1\right) $$

c) *Regulation of* glucose uptake and *mitochondrial pyruvate utilization*

MITOsym includes several signals where a pathway is regulated by the intermediate of its own pathway. These signals are used to describe the glucose uptake (${\mathrm{S}}_{{\mathrm{fb}}_{\mathrm{G}6\mathrm{P}2\mathrm{G}\mathrm{l}\mathrm{u}}}$) and mitochondrial pyruvate utilization (${\mathrm{S}}_{{\mathrm{fb}}_{\mathrm{mitoPyr}}}$ and ${\mathrm{S}}_{{\mathrm{fb}}_{\mathrm{mitoOX}}}$). The signals, generalized as Si_fb_, are computed as a Hill equation:

S4 $$ S{i}_{fb} = \left(K{m}^{Hill}+S{i}_{basal}^{Hill}\right)/\left(K{m}^{Hill}+S{i}_{current}^{Hill}\right) $$

where signals, Si_fb_, are based on the basal (*Si*_*basal*_) and current (*Si*_*current*_) states of its own intermediates.

d) *Regulation of mitochondrial ATP production by ATP levels*

Feedback inhibition has been reported for the mitochondria F_1_F_0_ ATPase (28), whereby extant ATP levels can modulate mitochondrial ATP synthesis. The signal *S*_fb _ ATP2mitoATP _ prod_ (Fig. 1) in MITOsym provides this type of feedback on the simulated mitochondria ATP production rate as shown in Table IV, Eq. EF6.

S5 $$ {S}_{{\mathrm{fb}}_{\mathrm{ATP}2{\mathrm{mitoATP}}_{\mathrm{prod}}}}=\left(2\times \frac{{\mathrm{BasalFlux}}_{{\mathrm{ATP}}_{\mathrm{total}}}}{{\mathrm{BasalFlux}}_{{\mathrm{ATP}}_{\mathrm{total}}}+{\mathrm{CurrentFlux}}_{{\mathrm{ATP}}_{\mathrm{total}}}}\right) $$

e) *ATP Regulation in glucose and galactose media*

The MITOsym model has been designed to compute cellular ATP levels when HepG2 cells are cultured in media containing either glucose or galactose as the primary fuel source. Galactose utilization has zero net ATP synthesized through glycolysis (29), as the ATP produced by glycolysis is offset by that required to phosphorylate galactose. Thus, ATP is completely supplied by mitochondria when cells are cultured in galactose. In MITOsym, the ATP production from glycolysis is set to zero when simulating cells cultured in galactose. A non-linear Hill equation is utilized to adjust simulated mitochondrial ATP production. The gradient-based signal (${\mathrm{S}}_{{\mathrm{fb}}_{\mathrm{MMP}2\mathrm{A}\mathrm{T}\mathrm{P}}}$) is given as:

S6 $$ {\mathrm{S}}_{{\mathrm{fb}}_{\mathrm{MMP}2\mathrm{A}\mathrm{T}\mathrm{P}}}=\frac{{\left({\mathrm{Km}}_{\mathrm{MMP}2\mathrm{A}\mathrm{T}\mathrm{P}}\right)}^{\mathrm{n}}}{\left({{\mathrm{Km}}_{\mathrm{MMP}2\mathrm{A}\mathrm{T}\mathrm{P}}}^n+{\left(\frac{\varDelta \varPsi {\mathrm{m}}_{\mathrm{basal}}}{\varDelta \varPsi {\mathrm{m}}_{\mathrm{current}}}\right)}^{\mathrm{n}}\right)}-\frac{{\left({\mathrm{Km}}_{\mathrm{MMP}2\mathrm{A}\mathrm{T}\mathrm{P}}\right)}^{\mathrm{n}}}{\left({{\mathrm{Km}}_{\mathrm{MMP}2\mathrm{A}\mathrm{T}\mathrm{P}}}^n+1\right)} $$

The Km and n parameter values in this equation were estimated by comparing simulated cellular ATP levels with those reported by Marroquin *et al.* (2). in galactose- or glucose-cultured HepG2 cells 24 h following treatment with FCCP or rotenone (Table IV, Eq. EF7).

For glucose media, the simulations revealed that ${\mathrm{S}}_{{\mathrm{fb}}_{\mathrm{MMP}2\mathrm{A}\mathrm{T}\mathrm{P}}}$ alone was not sufficient to describe total ATP generated from mitochondria (OXPHOS) and cytosol (glycolysis). The altered ATP production rates with low dose rotenone or FCCP produced simulated ATP levels in excess of those reported. As such, introduced into MITOsym was a second signal, ${\mathrm{S}}_{{\mathrm{Eff}}_{\mathrm{glycolysis}}}$, which reduces the cellular pyruvate-to-ATP ratio when glycolysis signals are up-regulated.

S7 $$ {\mathrm{S}}_{{\mathrm{Eff}}_{\mathrm{glycolysis}}}=1-\mathrm{K}\cdot \left(\frac{\varDelta {{\mathrm{S}}_{{\mathrm{fb}}_{\mathrm{ATP}2\mathrm{g}\mathrm{l}\mathrm{y}}}}_{\mathrm{current}}}{\varDelta {{\mathrm{S}}_{{\mathrm{fb}}_{\mathrm{ATP}2\mathrm{g}\mathrm{l}\mathrm{y}}}}_{\mathrm{basal}}}\times \frac{\varDelta {{\mathrm{S}}_{{\mathrm{fb}}_{\mathrm{ETC}2\mathrm{g}\mathrm{l}\mathrm{y}}}}_{\mathrm{current}}}{\varDelta {{\mathrm{S}}_{{\mathrm{fb}}_{\mathrm{ETC}2\mathrm{g}\mathrm{l}\mathrm{y}}}}_{\mathrm{basal}}}-1\right) $$

The coefficient, K, was first adjusted to simulate ATP levels consistent with those measured in HepG2 cells treated with low dose rotenone and FCCP while cultured in 25 mM glucose. K was subsequently refined by simultaneously adjusting it in concert with ${\mathrm{S}}_{{\mathrm{fb}}_{\mathrm{MMP}2\mathrm{A}\mathrm{T}\mathrm{P}}}$ to generate simulated ATP levels across the full dose range for both mitochondrial effectors. It was assumed that ATP utilization was consistent in all experimental settings.

## Results

### Effects of Media Glucose Concentrations on OCR and ECAR

**Fig. 2:**
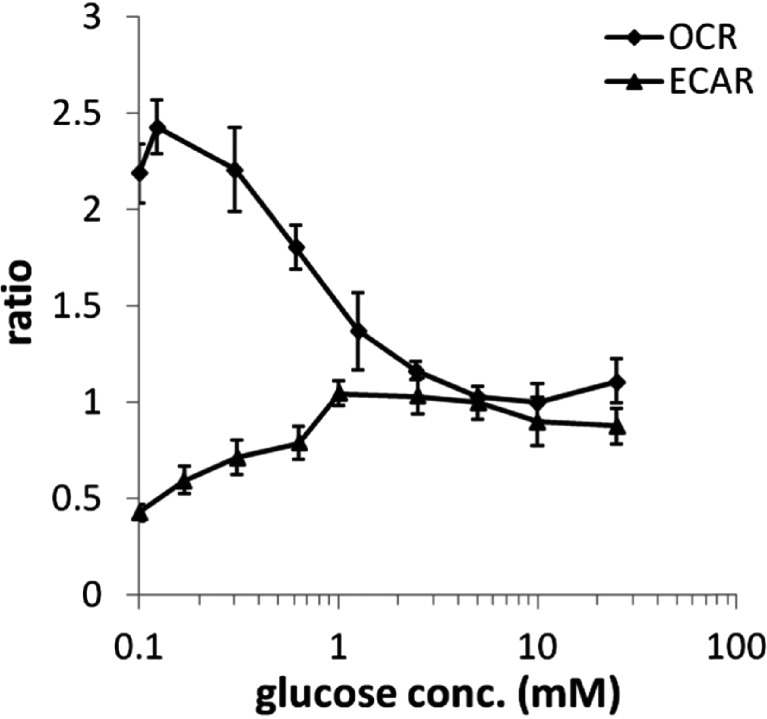
Measured basal OCR and ECAR response with increasing glucose concentration in HepG2 cells (range 0–25 mM glucose). Note that the displayed OCR and ECAR ratios were computed by normalizing by the measured values by those collected from the 5 mM glucose experiments.

**Fig. 3:**
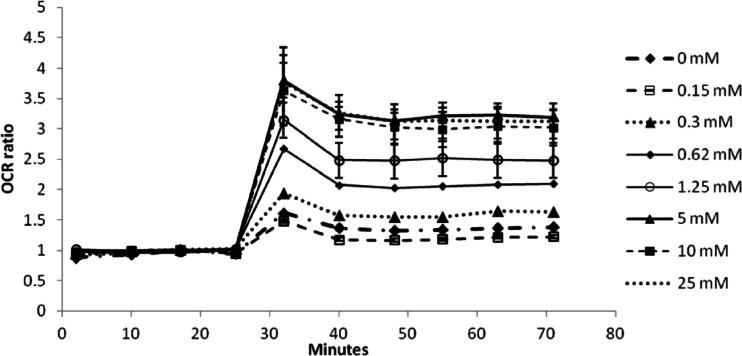
Measured spare respiratory capacity in HepG2 cells exposed to various media glucose concentrations (range 0–25 mM glucose). Cells were exposed to 3 uM FCCP at t = 25 min. The OCR values have been normalized by the values collected prior to FCCP exposure to compute the OCR ratio.

Effects of media glucose concentrations on oxygen consumption rates (OCR) and extracellular acidification rates (ECAR) were investigated in this study. OCR and the ECAR of HepG2 cells were measured in real-time, simultaneously, in an extracellular flux analyzer (Seahorse Bioscience, Billerica, MA) with various concentrations of glucose. The reserve capacity of the cells was measured by the addition of 3 μM FCCP, and the OCR and ECAR immediately prior to FCCP injection were used as the basal OCR and ECAR, respectively (see Method section for more details). In these experiments, OCR and ECAR both changed as media glucose concentrations increased from 0 to 25 mM (Figs. 2 and 3). OCR was highest when cells were cultured in 0 mM glucose and decreased towards a plateau as media glucose levels reached 5 mM; the plateau OCR was 2.5 fold lower than the value at 0 mM media glucose. In contrast, ECAR increased with glucose concentrations. The spare respiratory capacity following the administration of 3 μM FCCP increased with glucose levels and reached a plateau at 5 mM (Fig. 3).

**Fig. 4:**
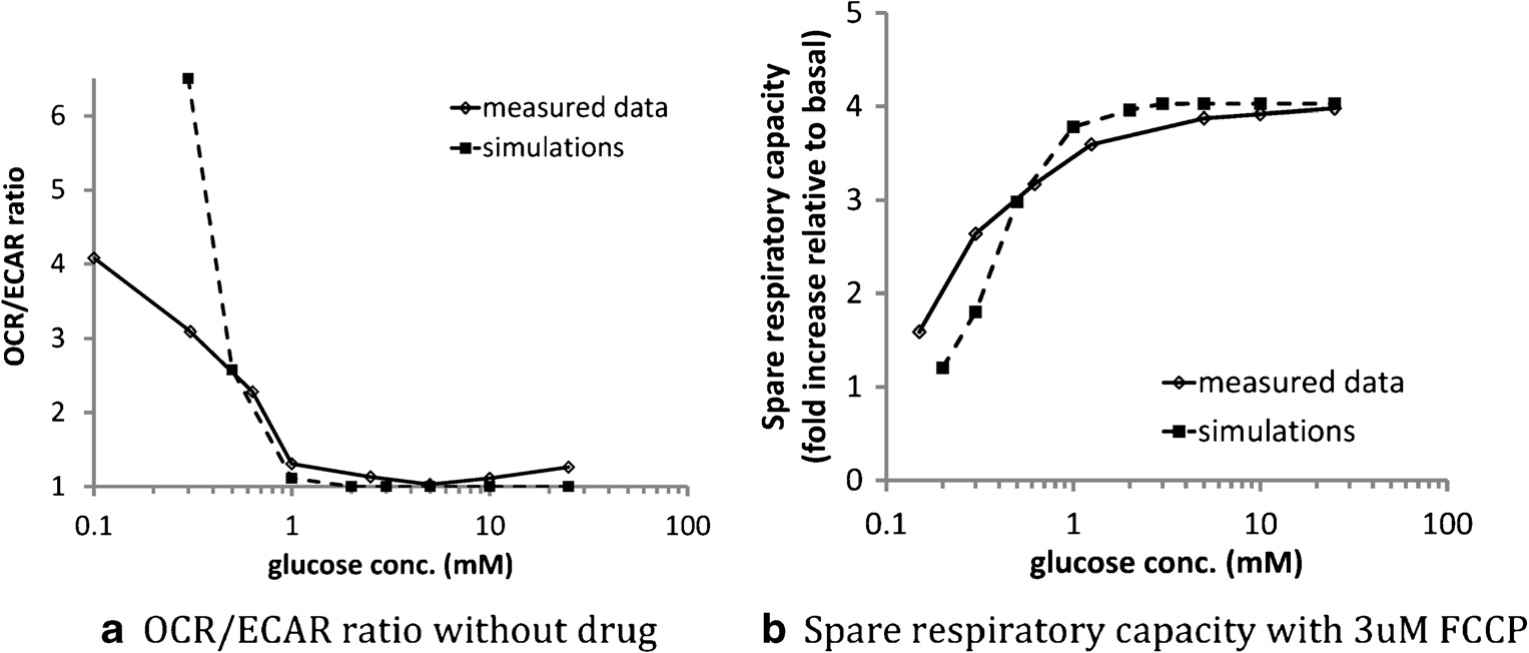
Comparison of the observed *vs.* simulated OCR/ECAR ratio (*left*) and spare respiratory capacity (*right*) in HepG2 cells exposed to various levels of media glucose (range 0–25 mM glucose). Note that the dependent values (Y-axis) are normalized by the values reported or simulated at 5 mM glucose condition.

These results were used to guide dynamics of glucose uptake and utilization in MITOsym as described in the Methods section. Low glucose media decreased G6P production rate (*Flux*_*prod*_^*G*6*P*^) which activated a feedback signal (${\mathrm{S}}_{{\mathrm{fb}}_{\mathrm{G}6\mathrm{P}2\mathrm{G}\mathrm{l}\mathrm{u}}}$) (Table V) to increase the flux of glucose 6 phosphate (*Eff* _ *Flux*_*prod*_^*G*6*P*^). At 1 mM glucose, the modulated glycolysis flux (*Eff* _ *Flux*_*prod*, *cellular*_^*pyr*^) is still lower than the flux estimated for 5 mM glucose. However, there was only a slight reduction in mitochondrial proton gradient and mitochondrial ATP production by the increased mitochondrial signal: ${\mathrm{S}}_{{\mathrm{fb}}_{\mathrm{mitoPyr}}}$, ${\mathrm{S}}_{{\mathrm{fb}}_{\mathrm{mitoOX}}}$ and ${\mathrm{S}}_{{\mathrm{fb}}_{\mathrm{H}\_\mathrm{Gradient}}}$ (Table V). The simulated OCR and ECAR profiles with increasing media glucose concentrations were similar to the measured data, as illustrated by the similarity in simulated and measured OCR/ECAR ratios (Fig. 4a). The simulated dose–response relationship between media glucose and respiratory capacity was also comparable with the measured data (Fig. 4b), showing an increase as glucose concentrations increased to a plateau when glucose ≥ 2 mM.

**Table V:** Bioenergetic Parameters and Properties Simulated in Various Conditions

		Basal value (Glucose)	Basal value (Galactose)	Rotonone 0.5 uM	FCCP 3 uM	Oligomycin 1 uM	
		5.5 mM 1 mM					
Intermediates							
G6P	[mM]	1.0	0.13	1.0	0.96	0.87	0.98
Pyruvate_cellular_	[mM]	5.0	4.64	4.7	7.91	4.95	6.95
Pyruvate_mito_	[mM]	5.0	4.89	4.7	8.12	4.11	6.70
ATP_cellular_	[mM]	4.0	3.95	3.88	1.97	3.41	NA
*ΔΨ*_*m*_		172.2	172.2	169.4	37.4	124.0	184.3
Calculated flux							
*Eff* _ *Flux*_*prod*_^*G*6*P*^	[mM/min]	1.87	1.76	2.13	2.56	4.19	2.35
*Eff* _ *Flux*_*prod*, *cellular*_^*pyr*^	[mM/min]	3.75	3.53	4.26	5.18	8.38	4.69
*Eff* _ *Flux*_*prod*_^*lac*^	[mM/min]	3.15	2.92	3.45	4.98	5.96	4.38
*Eff* _ *Flux*_*prod*, *mito*_^*pyr*^	[mM/min]	0. 6	0. 6	0.84	0.19	2.42	0.31
*Eff* _ *Flux*_*prod*_^*pyr*2*OX*^	[mM/min]	0. 6	0. 6	0.84	0.19	2.42	0.31
*Eff* _ *Flux*_*prod*_^*ETC*^	[mM/min]	0. 6	0. 6	0.84	0.19	2.42	0.31
*Eff* _ *Flux*_*mito*,*lowgradient*_^*ATP*^	[pmol ATP/min]	1000	999.6	1334.7	259.7	711.1	NA
*Eff* _ *Flux*_*prod*,*gly*_^*ATP*^	[pmol ATP/min]	337.2	320.3	0	403.1	430.8	NA
Pathway signal (normalized)							
${\mathrm{S}}_{{\mathrm{fb}}_{\mathrm{H}\_\mathrm{Gradient}}}$		1	1.02	1.38	4.17	4.17	0.19
${\mathrm{S}}_{{\mathrm{fb}}_{\mathrm{G}6\mathrm{P}2\mathrm{G}\mathrm{l}\mathrm{u}}}$		1	3.40	1.14	1.38	2.24	1.24
${\mathrm{S}}_{{\mathrm{fb}}_{\mathrm{ATP}2\mathrm{g}\mathrm{l}\mathrm{y}}}$		1	1	1.00	1.08	1.02	1.06
${\mathrm{S}}_{{\mathrm{fb}}_{\mathrm{ETC}2\mathrm{g}\mathrm{l}\mathrm{y}}}$		1	1	1.15	1.28	2.21	1.19
${\mathrm{S}}_{{\mathrm{fb}}_{\mathrm{mitoPyr}}}$		1	1.07	1.19	0.23	1.8	0.42
${\mathrm{S}}_{{\mathrm{fb}}_{\mathrm{mitoOX}}}$		1	1.07	1	0.07	1	2.56
${\mathrm{S}}_{{\mathrm{fb}}_{\mathrm{MMP}2\mathrm{A}\mathrm{T}\mathrm{P}}}$		1	1	0.97	0.88	0.92	1
${\mathrm{S}}_{{\mathrm{Eff}}_{\mathrm{glycolysis}}}$		1	0.99	NA	0.89	0.63	1
*S*_fb _ ATP2mitoATP _ prod_		1	1	1.4	1.18	1.05	1

### Simulation of Mechanism-Based Mitochondrial Responses to Specific Compounds

**Fig. 5:**
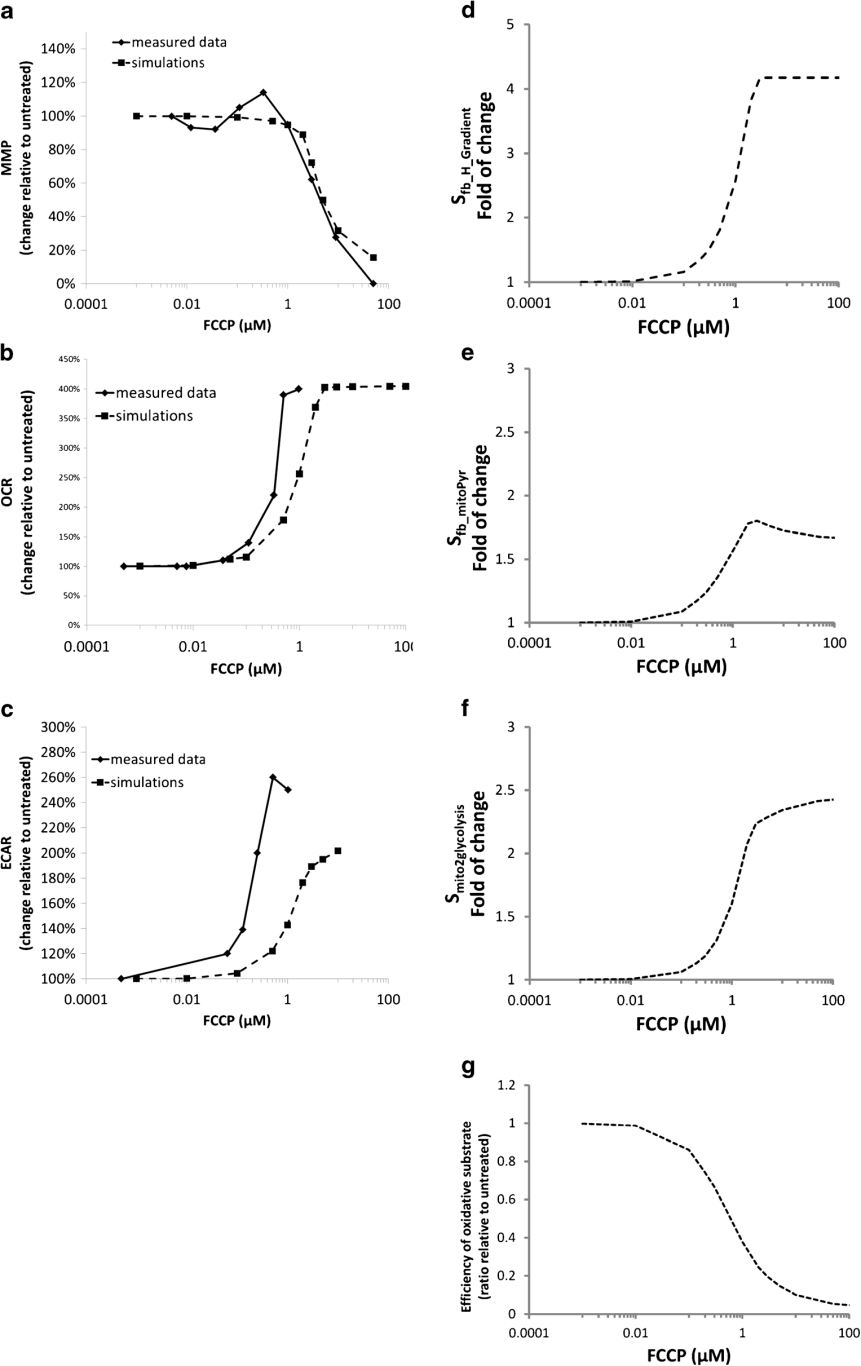
Comparison of the simulated and measured OCR, ECAR and mitochondria membrane gradient and the associated regulation signals.

**Fig. 6:**
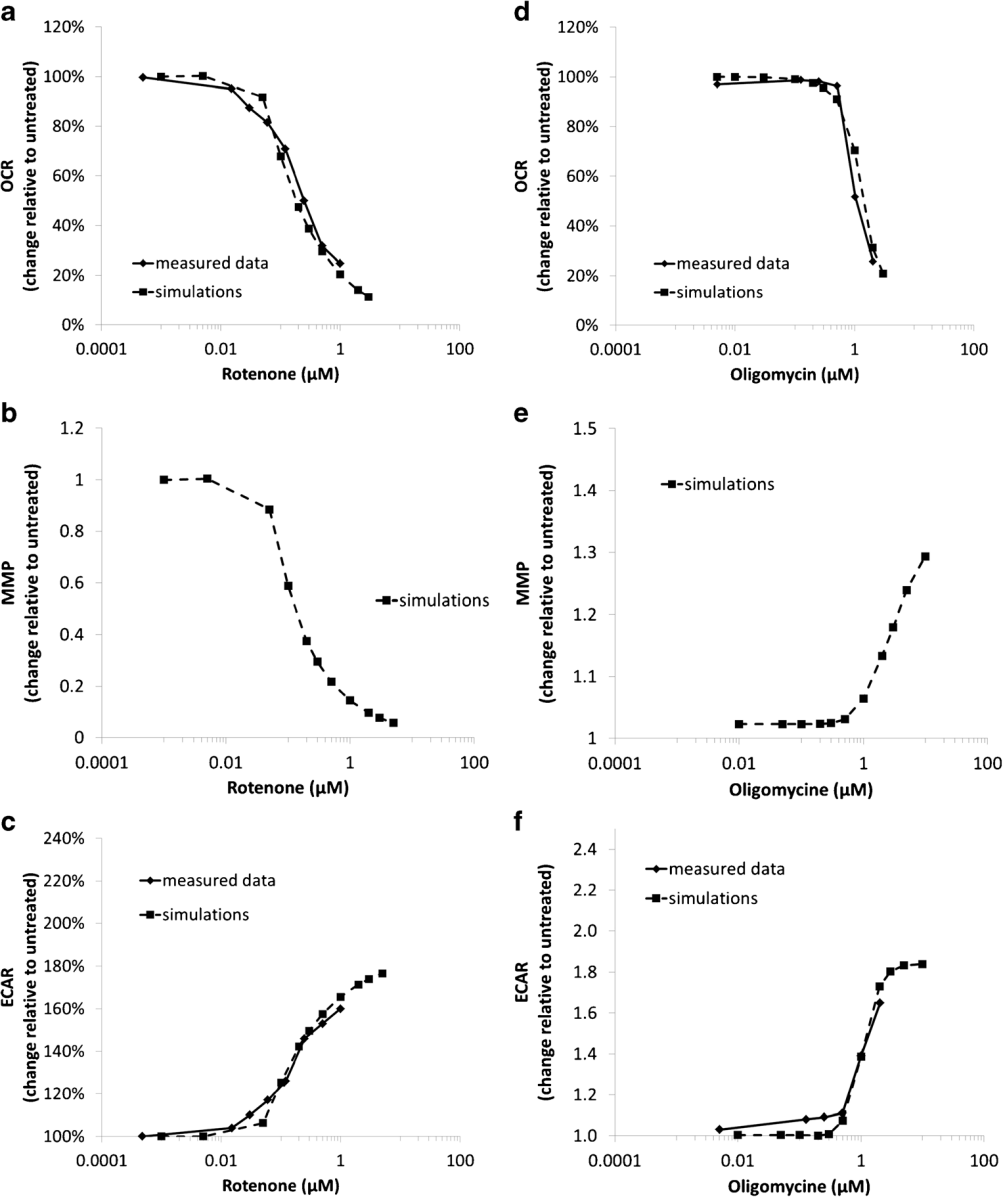
Simulation of OCR, ECAR and mitochondria membrane potential in response to rotenone and oligomycin. Comparisons to measured OCR and ECAR are also included.

Mechanism-specific disruptions of mitochondrial function were evaluated with three classic mitochondrial compounds in MITOsym. Rotenone was used to investigate the mitochondrial response to direct ETC complex I inhibition. FCCP was used to test respiratory capacity when compensating for the uncoupling of the proton gradient. Oligomycin was used to evaluate the mitochondrial response to inhibition of the F_1_F_O_ - ATPase. To examine the mechanism-specific effect of the exemplar compounds (rotenone, FCCP, and oligomycin), cellular respiration (OCR) and glycolysis (ECAR) were measured before and after addition of these compounds. The OCR and ECAR immediately prior to the compound injection were used as the basal OCR and ECAR, respectively, and were defined as 100% when generating the concentration-response curves for each compound. OCR and ECAR data for rotenone and FCCP(3) in addition to new data for oligomycin effects on OCR and FCCP effects on ΔΨm are listed in Appendix C and Figs. 5 and 6. FCCP, rotenone, and oligomycin all display a clear dose–response relationship for OCR and ECAR. However, the effects on OCR are compound specific. OCR was increased by 400% when FCCP ≥ 1 uM (Fig. 5b), while decreases in OCR are observed for both rotenone (Minimum OCR = 20% of initial value) and oligomycin (Minimum OCR = 26% of initial value) (Fig. 6a, d). ECAR was increased 150, 60 and 40% for 1 uM FCCP (Fig. 5c), rotenone (Fig. 6b), and oligomycin (Fig. 6e), respectively. Additionally, FCCP decreased the ΔΨm by 5% at 1 uM and by 65% at 3 uM (Fig. 5a).

These mechanism-specific data were used to construct a homeostatic control network in MITOsym consisting of various regulation signals (Fig. 1) to describe how the metabolic pathways interact with each other. The simulated bioenergetic outputs are the synchronized results of modulated metabolic fluxes which account for single or multi-step signals.

Using FCCP as an example, the increased uncoupling effects lead to reduced ΔΨm (Fig. 5a) and increases in ${\mathrm{S}}_{{\mathrm{fb}}_{\mathrm{H}\_\mathrm{Gradient}}}$ (Fig. 5d) and ETC activity (Fig. 5b). Increased ETC activity reduced the mitochondrial oxidative substrate ([Pyruvate_mito_]), and increased the flux of pyruvate entering mitochondria through increased ${\mathrm{S}}_{{\mathrm{fb}}_{\mathrm{mitoPyr}}}$ (Fig. 5e). In parallel, the decreased ΔΨm and increased ETC activity together up-regulated glycolysis signals (Fig. 5f), which led to ECAR increases (Fig. 5c). The simulated ΔΨm and OCR are comparable with the measured data. However, the simulated ECAR was underestimated. One thing to note is that the magnitude of change in MITOsym relating the feedback signal for glycolysis (${\mathrm{S}}_{{\mathrm{fb}}_{\mathrm{mito}2\mathrm{glycolysis}}}$) (2.5X) was greater than the simulated ECAR change (2.1X) (Fig. 5c *vs.* 6f). This is due to the fact that simulated ECAR is computed only from lactate flux, while glycolysis provides pyruvate for both lactate release and mitochondria pyruvate uptake.

The simulated OCR and ECAR outputs are also in agreement with the measured data for rotenone and oligomycin (Fig. 6). By design, a non-linear relationship between ETC flux (hence, OCR) and ΔΨm are predicted for rotenone (Fig. 6b). Our simulation results predicted an increase in ΔΨm (Fig. 6e) for oligomycin (despite the decrease in OCR, Fig. 6d) as a consequence of the increased ΔΨm eliciting a decrease in the magnitude of the feedback signal ${\mathrm{S}}_{{\mathrm{fb}}_{\mathrm{H}\_\mathrm{Gradient}}}$.

The metabolic fluxes and the relative intermediate concentrations computed with MITOsym provide additional information on the predicted integrated response to mitochondrial effectors. Simulations with MITOsym indicate that 0.5 uM rotenone would inhibit 70% of ETC activity in HepG2 (as illustrated by OCR in Fig. 4a), cause the accumulation of cellular and mitochondria pyruvate, a decrease in ΔΨm, and an associated reduction of ATP (Table V). Moreover, these changes elicited further alterations in the glycolysis flux (*Eff*_*Flux*_ _*prod*, *cellular*_^*pyr*^, Table V), motivated by increases in the feedback signals, (${\mathrm{S}}_{{\mathrm{fb}}_{\mathrm{ETC}2\mathrm{g}\mathrm{l}\mathrm{y}}}$ and ${\mathrm{S}}_{{\mathrm{fb}}_{\mathrm{ATP}2\mathrm{g}\mathrm{l}\mathrm{y}}}$).

For 3 uM FCCP, the model predicted a 30% reduction of *ΔΨ*_*m*_, despite the 417% increased of ETC activity (*Eff* _ *Flux*_*prod*_^*ETC*^) relative to untreated values. The augmented ETC activity was largely due to the effects of the feedback signal (${\mathrm{S}}_{{\mathrm{fb}}_{\mathrm{H}\_\mathrm{Gradient}}}$), which increased substantially in response to the reduced ΔΨm (Table V). The predicted response to 1 uM oligomycin included an increase in ΔΨm, a 50% reduction in ETC activity, OCR, and an accumulation of cellular and mitochondria pyruvate (Table V). The reduced ETC activity was primarily the result of two feedback signals, (${\mathrm{S}}_{{\mathrm{fb}}_{\mathrm{mitoOX}}}$ and ${\mathrm{S}}_{{\mathrm{fb}}_{\mathrm{H}\_\mathrm{Gradient}}}$).

### Effects of Consecutive Administration of Mitochondria Effectors on OCR

**Fig. 7:**
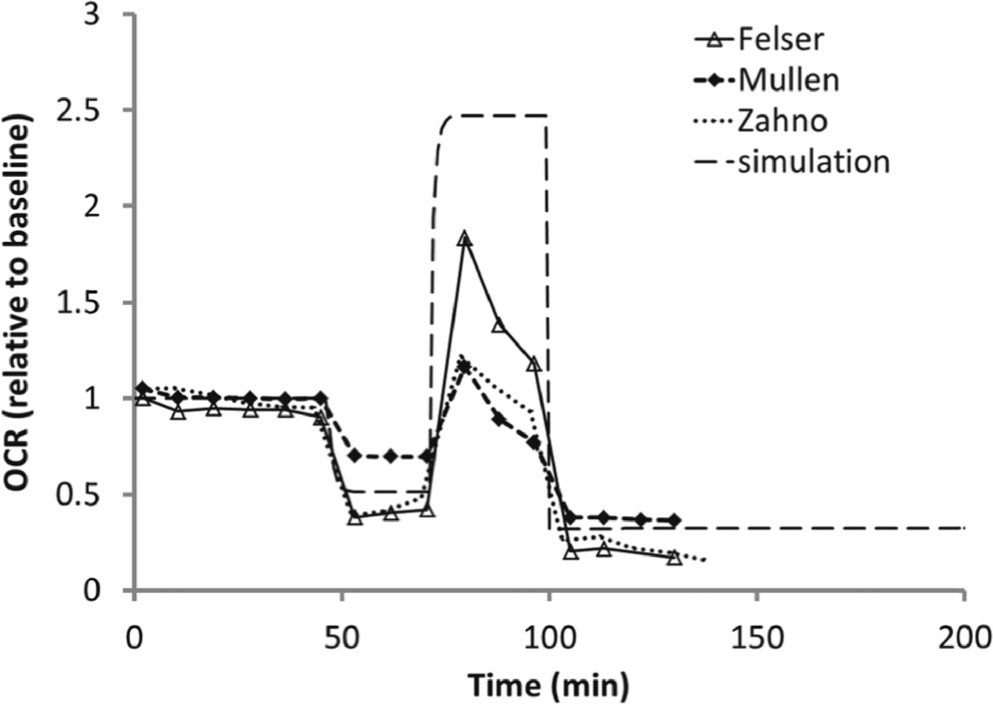
Comparison of the simulated time-series OCR results with data reported by three independent studies in HepG2 cells following a “stress-test” protocol (1 μM of oligomycin, FCCP, and rotenone, added sequentially) (Ferrick *et al.*, 2008)

We next investigated whether MITOsym could simulate effects of consecutive administration of the mitochondria effectors, oligomycin, FCCP, and rotenone on OCR. As illustrated in Fig. 7, 1 uM of oligomycin, FCCP, and rotenone were sequentially administered. The measured OCR decreased 50–70% in response to 1 μM oligomycin, increased to 20 ~ 100% above basal levels after 1 μM FCCP, and decreased to 20–35% of basal after 1 μM rotenone, as reported previously (9–11). This experimental protocol was simulated in MITOsym (Fig. 7) using the same parameters as were used to generate the response to each individual compound (Figs. 5 and 6). Therefore, these simulations serve as a validation of the framework of MITOsym. The simulation results compare well with the measured experimental data, given the amount of reported variability using this experimental protocol.

### Effects of Rotenone or FCCP on ATP levels in Simulated HepG2 Cells Cultured in Glucose or Galactose

MITOsym was extended beyond simulations of respiration to predictions of ATP levels in cultured HepG2 cells. The relative contributions to ATP from mitochondria and glycolysis were captured in simulations of cells cultured in media containing glucose or galactose, as galactose does not provide net ATP synthesis *via* glycolysis. For these simulations, the model settings were similar except for the net ATP production from glycolysis is zero and mitochondria ATP production (${\mathrm{S}}_{{\mathrm{Eff}}_{\mathrm{MMP}2\mathrm{A}\mathrm{T}\mathrm{P}}}$) is less efficient for the galactose media condition. For ATP estimation, ATP-dependent (1st order) reduction in ATP utilization was employed; and an additional glycolysis-ATP adjustment signal (${\mathrm{S}}_{{\mathrm{Eff}}_{\mathrm{glycolysis}}}$) was included for glucose media (Table IV, EF8 and Table V). Moreover, media-specific parameters to describe mitochondrial-ATP efficiency (${\mathrm{S}}_{{\mathrm{Eff}}_{\mathrm{MMP}2\mathrm{A}\mathrm{T}\mathrm{P}}}$) were determined based on comparison with measured and published ATP data (2) as described in the Methods section. Oligomycin-based changes to ATP levels were not included in these simulations due to the data disparity (see Discussion).

Simulated ATP levels reached a new steady state in less than 2 h when cells switched from glucose to galactose media. The simulated basal, untreated ATP concentrations for glucose and galactose media conditions are within 5% of each other (Table V), a finding consistent with published ATP data. Simulations of HepG2 cells cultured in galactose indicated that there was a 40% increase of mitochondrial ATP synthesis (*S*_fb _ ATP2mitoATP _ prod_), a 13% increase in glycolysis flux (*Eff* _ *Flux*_*prod*, *cellular*_^*pyr*^), and a 40% increase in ETC activity (*Eff* _ *Flux*_*prod*_^*ETC*^) (Table V) relative to simulations of HepG2 cells in glucose media.

**Fig. 8:**
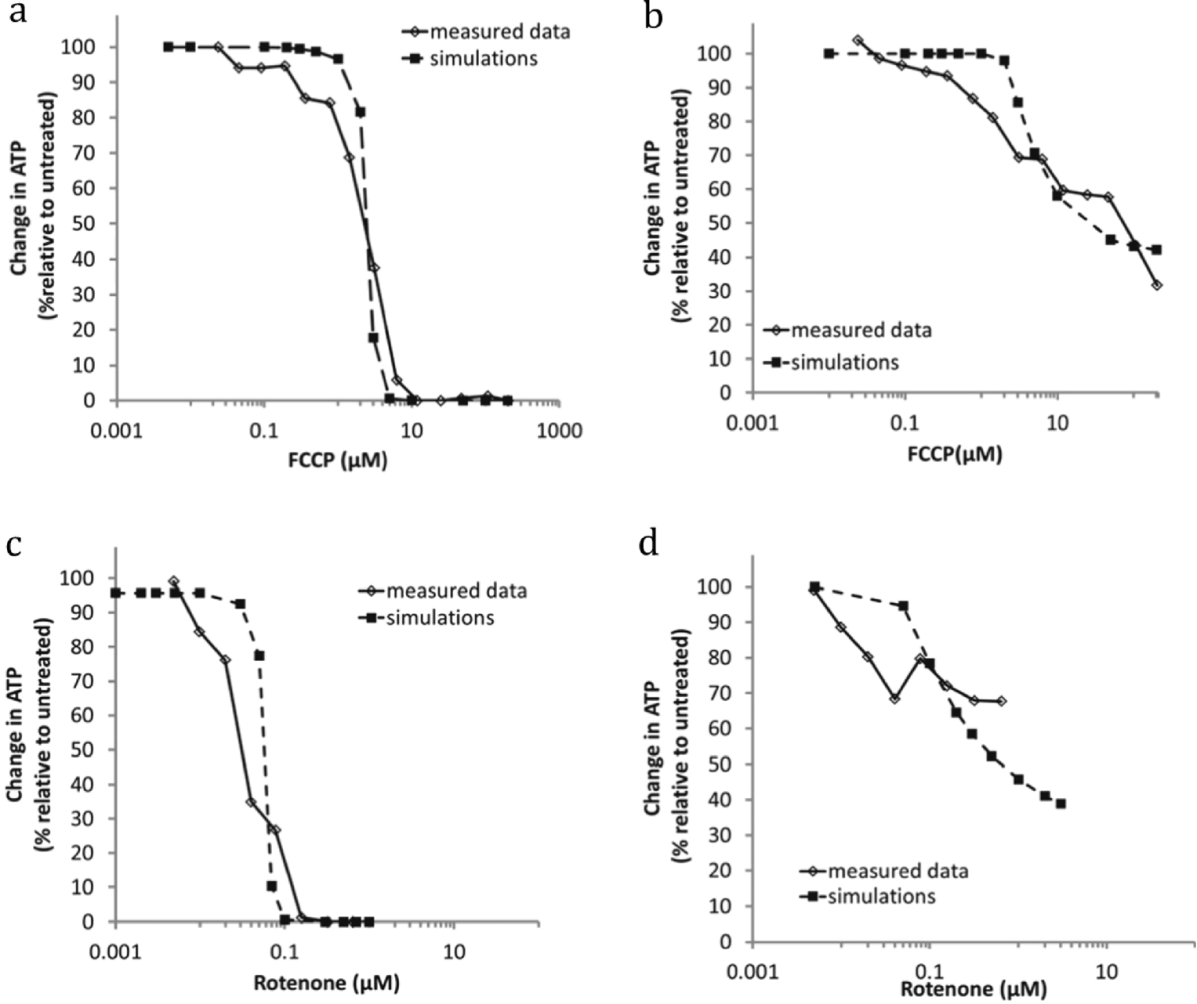
Comparison of the measured *versus* the simulated change in cellular ATP exposed to FCCP or rotenone when HepG2 cells were cultured in media containing galactose (10 mM) or glucose (25 mM). (**a**) FCCP-ATP dose response in galactose media; (**b**)Rotenone-ATP dose response in galactose media; (**c**)FCCP-ATP dose response in glucose media; (**d**)Rotenone-ATP dose response in glucose media.

There was good agreement between the simulated ATP levels and the measured data across a dosing range of rotenone and FCCP in HepG2 cells cultured in either galactose or glucose media (Fig. 8). In the simulations of HepG2 cells in glucose media, the pyruvate to ATP ratio appeared to be reduced with increased glycolytic activity (*i.e.*, at higher rotenone or FCCP exposure). It is estimated that contribution from glycolysis to ATP in simulations with 0.5 uM rotenone and 3 uM FCCP is 40 and 38% respectively, compared to 25% in the basal condition. Moreover, with 3 uM FCCP, glycolysis (*Eff* _ *Flux*_*prod*,*cellular*_^*pyr*^) was predicted to increase 220% while the simulated ${\mathrm{S}}_{{\mathrm{Eff}}_{\mathrm{glycolysis}}}$ is 63% of baseline (Table V). The low ΔΨm when HepG2 were exposed to 3 uM FCCP also likely invoked the mitochondrial utilization of ATP. The simulated net mitochondria ATP production rates (including possible contributions from hydrolysis) were observed to be lower in the galactose (77% reduction) vis a vis glucose (8% reduction) media conditions. Overall, the good agreement between the measured and simulated ATP concentrations confirms that MITOsym properly captures ATP production and utilization dynamics.

## Discussion

Described herein is a mathematical model of hepatocellular respiration and bioenergetics called MITOsym that has been developed with the purpose of supporting drug screening efforts towards reducing drug toxicity. Mitochondria-based toxicity and dysfunction has been suggested to contribute to the hepatotoxicity observed with multiple drugs. As such, cell-based measurements of respiration using instruments such as the Seahorse Biosciences XF analyzers (6) have been increasingly used in the drug development process. MITOsym has been designed to align with the OCR and ECAR measures from these instruments in predicting hepatocellular changes in ATP based on alterations in mitochondrial and glycolytic function. This model includes mathematical representations of the essential biochemical components of mitochondrial and bioenergetic function, particularly in the *in vitro*, cultured HepG2 cell environment. Data from experimental procedures like those described in the examples herein, as well as published data, can serve to calibrate the dynamics of these interactions. Moreover, MITOsym has been designed to support predictions of *in vivo* hepatotoxicity in mice, rats, dogs, and/or humans using DILIsym® (27).

### The Dependence of HepG2 Bioenergetics on Media Glucose

Glucose uptake and its contributions to bioenergetics appear to be cell-type or tissue-type dependent. We observed a dependence of OCR and ECAR on media glucose levels in HepG2 cells. In support of these results, Hue *et al.* (30) reported increases in rat hepatocyte glycolysis and lactate release in cells exposed to 0–50 mM media glucose. Others have also reported a glucose dependency for respiration in various tissues, including human islets and porcine annulus fibrosus.

It appears that the saturation level for glucose concentrations on HepG2 cell uptake, glycolysis, and cellular respiration differs depending on the metabolic state of the cell. Our results indicate that plateau OCR and ECAR levels were achieved at ≥ 3 mM media glucose when the HepG2 cells were in their basal state. Upon the introduction of substantial levels of FCCP and its increased respiratory burden due to the uncoupling effects, the plateau OCR was observed at ≥ 5 mM media glucose. It is likely that the increases in respiration when mitochondria are uncoupled require a higher level of oxidative substrate than is needed in the basal state. MITOsym captures the dose-dependency of media glucose on OCR and ECAR in both the basal and the uncoupled states in HepG2 cells (Fig. 4).

### The Dynamics and Regulation of Mitochondrial Membrane Potential and Respiration

Mitochondria are able to adjust ETC activity to accommodate changes to the mitochondrial membrane potential. We observed a reduction in ΔΨm when HepG2 were treated with ≥1 μM FCCP, yet Nadanaciva *et al.* (2012)(3) reported OCR increases at ≥0.1 μM (Fig. 5). The increased OCR is due to an adaptive increase in ETC flux in an attempt to preserve ΔΨm in the presence of the uncoupling effect of FCCP. The loss of ΔΨm at ≥1 μM FCCP is likely due to inadequate provision of substrate to sustain the increase in ETC activity. Others have reported substantial reductions in ΔΨm in the presence of ≥1 μM FCCP (23,31) and increases in respiration ≥0.1 μM FCCP (31).

The OCR reduction we observed when HepG2 cells were treated with increasing concentrations of oligomycin (Fig. 6d–f) is also likely linked to changes in ΔΨm. While not measured in our study, oligomycin has been previously shown to increase ΔΨm in rat hepatocytes (22,24), and this is thought to be due to mitochondria F_1_F_O_ -ATPase inhibition and associated reductions in proton uptake (32). Reduced proton flux likely reduces ETC complex activity as well, providing a rationale for our observed dose-related OCR decreases. The degree of OCR reductions is consistent with what has been reported previously.

We captured this bidirectional relationship between ΔΨm and ETC activity (as measured by OCR) in MITOsym. The ΔΨm and OCR data from the experiments with FCCP and oligomycin were used to help quantify this relationship and identify the appropriate parameter values. A decrease in ΔΨm generated a signal (${\mathrm{S}}_{{\mathrm{fb}}_{\mathrm{H}\_\mathrm{Gradient}}}$) that increases ETC activity; the magnitude of this signal was based on the FCCP data (Fig. 5). Conversely, ${\mathrm{S}}_{{\mathrm{fb}}_{\mathrm{H}\ \mathrm{Gradient}}}$ decreases ETC activity as ΔΨm increases. Oligomycin data were used to calibrate the signal for this purpose. One thing to note is that we observed a decrease in ΔΨm at higher oligomycin concentrations (or longer incubation times) in our laboratory (data not shown). Descriptions of this inverse oligomycin-gradient relationship at higher doses could be included in the MITOsym in the future. However, simulations revealed that ${\mathrm{S}}_{{\mathrm{fb}}_{\mathrm{H}\_\mathrm{Gradient}}}$ alone was not sufficient to accurately capture the ΔΨm and ETC activity relationship. As such, we introduced a second signal, ${\mathrm{S}}_{{\mathrm{fb}}_{\mathrm{mitoPyr}}}$, which captured the feedback on mitochondrial substrate uptake based on changes in ETC activity (Fig. 6e). The rationale for this signal is that reductions in ETC activity (such as with oligomycin or rotenone) would cause an accumulation of oxidative substrate within mitochondria; this accumulation of substrate would further influence mitochondrial uptake of substrate. The combined signals work to restrict OCR when FCCP is administered following oligomycin (Fig. 6) *vs.* when FCCP is administered alone (Fig. 5). The difference is a result of feedback from accumulation of mitochondrial oxidative substrate (${\mathrm{S}}_{{\mathrm{fb}}_{\mathrm{mitoPyr}}}$) due to oligomycin.

### The Dynamics and Regulation of Glycolysis

It has been repeatedly observed that glycolysis rates increase during times of mitochondrial duress (3,4,16). The increased extra-mitochondrial ATP production *via* glycolysis can offset the drug-induced decrement in mitochondrial ATP production (2,4).

This dynamic interplay between mitochondrial impairment and glycolysis rates has been included in MITOsym. In MITOsym, glycolysis is described as the formation of G6P and the subsequent conversion to pyruvate. Glycolysis has been shown to be regulated by ATP levels (17), and ATP was employed as the signal between mitochondria and glycolysis in initial simulations. However, neither ATP concentrations, ΔΨm, nor the rate of change of either were sufficient descriptors for the activation of glycolysis in HepG2 cells treated with FCCP, rotenone, or oligomycin. It is possible that additional descriptions of metabolic intermediates such as AMP or ADP could provide a better quantitative description for glycolysis as demonstrated in Schmitz *et al.* which used AMP/ADP concentration and calcium-calmodulin-PFK complex to explain the glycolysis regulation in muscle tissues (33). In MITOsym, however, a more empirical approach was used to derive the dynamic between glycolysis and mitochondrial oxidation. Glycolysis increases are regulated jointly by changes of total ATP and ETC activity. Simulated ECAR results are in good agreement with the measured data collected for rotenone and oligomycin (Fig. 6) but underestimates ECAR values for FCCP (Fig. 5). The need for two signals to regulate the link between mitochondrial function and glycolysis is made apparent when comparing the response to FCCP vs rotenone. With FCCP, there is a 150% increase in ECAR while there is only a 10% reduction in ΔΨm (Fig. 5). A similar ΔΨm reduction for rotenone elicits only a 10% ECAR increase, however (Fig. 6).

### Regulation of ATP

A critical index of mitochondrial dysfunction and risk for tissue injury is cellular ATP concentrations. Both utilization and production contribute to ATP levels. ATP utilization in MITOsym is a concentration dependent, first-order process. Decreases in ATP production are met with a reduction in ATP utilization, acting to preserve cellular function and avoid ATP depletion and the resultant necrosis (34). This modeling approach is consistent with what has been described previously (14).

ATP production receives contributions from both mitochondrial OXPHOS and cytosolic glycolysis. Cells cultured in media containing glucose receive contributions from both pathways, while cells cultured in galactose only rely on mitochondrial ATP production (2,29,35). We utilized data from HepG2 cells cultured in galactose- or glucose-containing media to establish the dynamics of mitochondrial ATP production. Simulation results showed there were increases in mitochondrial ATP synthesis, glycolysis flux and ETC activity for HepG2 cells cultured in galactose media (Table V). These energy substrate-dependent changes are consistent with data in various cells types (29,35,36).

The simulated changes in ATP levels with MITOsym were generally consistent with the reported levels in HepG2 cells treated with rotenone and FCCP in both galactose and glucose media (2). For example, ATP levels were not fully depleted in the presence of large amounts of FCCP or rotenone in simulations of HepG2 cells cultured in glucose media (Fig. 8c,d); conversely, simulations with galactose media indicated complete loss of ATP (Fig. 8a,b). Glycolysis was able to partially preserve ATP levels despite the loss of mitochondrial ATP production.

Close inspection of the dose–response curves for both FCCP and rotenone indicate that the simulations overestimate ATP levels at lower exposures relative to the measured data. Using 1 μM FCCP as an example, the increases in OCR and ECAR and the minor decrease in ΔΨm (Fig. 6) indicate mitochondrial ATP production was preserved. Based on the mitochondrial ATP production rates for the change in ΔΨm (5% reduction) and the cytosolic ATP production rates based on the measured ECAR (increased 100%), cellular ATP levels were also predicted to be preserved in galactose media but increased for glucose media. Marroquin *et al.*(2), however, reported a 20 and 15% reduction in ATP in HepG2 cells when cultured in galactose and glucose media with 1uM FCCP respectively. It is possible that the mild uncoupling elicited an increase in ATP utilization. It is also possible that there are time-related differences, since OCR and ECAR are measured within 2 h after FCCP treatment; while the ATP levels were measured at 24 h.

Oligomycin inhibition of the F_1_F_O_ - ATPase leads to reductions of ATP levels, decreases in OCR, and increases in ECAR (2,3). Interestingly, there are differences in the dose–response relationships between oligomycin *vs.* ATP and oligomycin *vs.* OCR. While MITOsym agrees well with both OCR and ECAR measured data following oligomycin treatment (Fig. 6) this is not the case with ATP levels (not shown). OCR inhibition plateaus with approximately 1 μM oligomycin in both simulations and measured data, but the measured depletion of ATP after 24 h of treatment is apparent with ≥0.01 μM oligomycin in HepG2 (2) and rat hepatocytes (34). We were unable to simulate similar reductions in ATP in MITOsym when oligomycin concentrations were that low (≥0.01 μM). Given the disparity in the measured exposure-response relationships, we have elected to optimize the oligomycin effects based on OCR and ECAR rather than the ATP data. Future versions of MITOsym may include both effects.

### Comparisons with Previous Models of Mitochondrial Function

Several previous reports of mathematical models of mitochondrial bioenergetics have been reported over time, including very detailed, thermodynamic-based models of isolated mitochondria (37–39). MITOsym, however, is focused on simulating mitochondrial function and dysfunction in a cell-based environment. MITOsym included essential biochemical components of mitochondria and bioenergetic function and has been designed to align with measurements of cellular respiration (OCR) and glycolysis (ECAR). Thus, some of the biochemical detail included in these previous models (*e.g.*, TCA cycle, calcium flux) has been excluded from MITOsym.

Hepatocyte modeling approaches similar to MITOsym have been reported previously by Ainscow *et al.* for primary rat hepatocytes and Dash *et al.* for skeletal muscle. With a control analysis approach, Ainscow *et al.* (14) described the control pattern between glycolysis, oxidative phosphorylation and ATP consumption. Each factor influences its own flux as well as the movement through the other metabolic blocks including glycogen breakdown, NADH oxidation, and proton leak (14). Dash *et al.* (40) investigated the regulation of metabolic processes during exercise in skeletal muscle. Included in this model are both glucose and fatty acid substrates, as skeletal muscle can utilize either substrate *in vivo*. One advancement that MITOsym offers over these previous modeling efforts is the ability to simulate the integrated bioenergetic response to multiple mitochondrial disruptors that have been co-administered (*i.e.*, oligomycin+FCCP+rotenone, Fig. 7).

MITOsym has several limitations that should be noted. It does not currently include contributions from both glucose and fatty acids to mitochondrial ATP production. MITOsym was designed to exclude fatty acids, consistent with most HepG2 and hepatocyte experimental protocols. Another limitation is that MITOsym does not discretely represent different mitochondrial ETC enzyme complexes, thereby limiting the ability to discern different ETC complex inhibitors from one another. Another limitation is the lack of published data indicating changes in cellular ATP levels during the same time frame as OCR and ECAR measurements (1–2 h after drug exposure). Rather, the preponderance of the ATP data for HepG2 was available after 24 h of exposure. Adaptations between 2 and 24 h may explain some of the differences between simulated ATP and measured data (Fig. 8). It should also be pointed out that mitochondrial turnover or adaptive biogenesis is not included, as these changes typically occur 24–48 h after injury. Finally, the current version of MITOsym may not accurately predict responses in primary hepatocytes due to differences between hepatocytes and HepG2 cells. Future versions of MITOsym will include some of the unique metabolic characteristics of primary hepatocytes and enable predictions of primary human and primary rat hepatocyte responses. Additional advancements may be made to address the other issues described here as well.

## Conclusion

MITOsym helps support the evaluation of compounds in drug development in several ways. The outputs have been designed to align with specific measurements of mitochondrial function using widely used laboratory instruments (*e.g.*, Seahorse XF analyzers). The cell-based measurement of respiration (and the modeling thereof) includes extra-mitochondrial adaptations. MITOsym includes several of these adaptive elements, such as the increase in glycolysis (as measured by ECAR) when mitochondrial function is compromised. MITOsym can also help with the interpretation of OCR and ECAR data, as it requires specific mechanistic elements to be defined when generating simulations of *in vitro* experiments. These mechanistic hypotheses can be confirmed when simulation results align with the measured data. Moreover, MITOsym can be used to identify compound-specific mitochondrial dysfunction parameter values that can be subsequently used in DILIsym® to predict *in vivo* hepatotoxicity based on *in vitro* compound data (8).

While this is best applied to the evaluation of hepatotoxicity in the version of MITOsym described in this report, it is also possible that the model could be adapted to other cell types. One such example could be adjusting the parameters to represent mitochondria and cellular function in neurons to support the development of treatments for Parkinson’s disease. Recent findings implicate mitochondrial dysfunction as a key molecular mechanism compromising dopamine neuronal function and survival, and as an underlying cause of pathogenesis in both sporadic and familial Parkinson’s disease.

It is our hope that MITOsym will indeed contribute to reducing the incidence of DILI. In particular, we anticipate that MITOsym can help reduce the resource burden associated with screening compounds for DILI risk. Simulations can reduce the number of subsequent wet lab experiments and potentially provide increased experimental focus. Also, as described above, modeling can provide users with a greater understanding of available data. MITOsym has been designed to represent the dynamics associated with disruptions in cell-based *in vitro* environments, and it will be partnered with DILIsym® to translate these *in vitro* results to predictions of *in vivo* hepatotoxicity risk in future studies.

## Electronic supplementary material

Below is the link to the electronic supplementary material.

ESM 1 (DOCX 37 kb)

## References

[1] Suk KT, Kim DJ (2012). Drug-induced liver injury: present and future. Clin Mol Hepatol.

[2] Marroquin LD, Hynes J, Dykens JA, Jamieson JD, Will Y (2007). Circumventing the Crabtree effect: replacing media glucose with galactose increases susceptibility of HepG2 cells to mitochondrial toxicants. Toxicol Sci Off J Soc Toxicol.

[3] Nadanaciva S, Rana P, Beeson GC, Chen D, Ferrick DA, Beeson CC (2012). Assessment of drug-induced mitochondrial dysfunction *via* altered cellular respiration and acidification measured in a 96-well platform. J Bioenerg Biomembr.

[4] Brand MD, Nicholls DG (2011). Assessing mitochondrial dysfunction in cells. Biochem J.

[5] Hynes J, Marroquin LD, Ogurtsov VI, Christiansen KN, Stevens GJ, Papkovsky DB (2006). Investigation of drug-induced mitochondrial toxicity using fluorescence-based oxygen-sensitive probes. Toxicol Sci Off J Soc Toxicol.

[6] Ferrick DA, Neilson A, Beeson C (2008). Advances in measuring cellular bioenergetics using extracellular flux. Drug Discov Today.

[7] Wu M, Neilson A, Swift AL, Moran R, Tamagnine J, Parslow D (2007). Multiparameter metabolic analysis reveals a close link between attenuated mitochondrial bioenergetic function and enhanced glycolysis dependency in human tumor cells. Am J Physiol Cell Physiol.

[8] Howell BA, Yang Y, Kumar R, Woodhead JL, Harrill AH, Clewell HJ (2012). In vitro to in vivo extrapolation and species response comparisons for drug-induced liver injury (DILI) using DILIsym^TM^: a mechanistic, mathematical model of DILI. J Pharmacokinet Pharmacodyn.

[9] Felser A, Blum K, Lindinger PW, Bouitbir J, Krähenbühl S (2013). Mechanisms of hepatocellular toxicity associated with dronedarone–a comparison to amiodarone. Toxicol Sci Off J Soc Toxicol.

[10] Zahno A, Brecht K, Morand R, Maseneni S, Török M, Lindinger PW (2011). The role of CYP3A4 in amiodarone-associated toxicity on HepG2 cells. Biochem Pharmacol.

[11] Mullen PJ, Zahno A, Lindinger P, Maseneni S, Felser A, Krähenbühl S (2011). Susceptibility to simvastatin-induced toxicity is partly determined by mitochondrial respiration and phosphorylation state of Akt. Biochim Biophys Acta.

[12] Nissim I, Horyn O, Nissim I, Daikhin Y, Wehrli SL, Yudkoff M (2012). Effects of a glucokinase activator on hepatic intermediary metabolism: study with 13C-isotopomer-based metabolomics. Biochem J.

[13] Hafner RP, Brown GC, Brand MD (1990). Analysis of the control of respiration rate, phosphorylation rate, proton leak rate and protonmotive force in isolated mitochondria using the “top-down” approach of metabolic control theory. Eur J Biochem FEBS.

[14] Ainscow EK, Brand MD (1999). Top-down control analysis of ATP turnover, glycolysis and oxidative phosphorylation in rat hepatocytes. Eur J Biochem FEBS.

[15] Zoratti M, Petronilli V (1985). Multiple relationships between rate of oxidative phosphorylation and delta microH in rat liver mitochondria. FEBS Lett.

[16] Dykens JA, Jamieson JD, Marroquin LD, Nadanaciva S, Xu JJ, Dunn MC (2008). In vitro assessment of mitochondrial dysfunction and cytotoxicity of nefazodone, trazodone, and buspirone. Toxicol Sci Off J Soc Toxicol.

[17] Berg JM, Tymoczko JL, Stryer L. The glycolytic pathway is tightly controlled [Internet]. 2002 [cited 2013 Sep 19]. Available from: http://www.ncbi.nlm.nih.gov/books/NBK22395/.

[18] Maier K, Hofmann U, Reuss M, Mauch K, Niebel A, Vacun G (2008). Identification of metabolic fluxes in hepatic cells from transient 13C-labeling experiments: Part I. Experimental observations. Biotechnol Bioeng.

[19] Okamoto T, Kanemoto N, Ban T, Sudo T, Nagano K, Niki I (2009). Establishment and characterization of a novel method for evaluating gluconeogenesis using hepatic cell lines, H4IIE and HepG2. Arch Biochem Biophys.

[20] http://www.seahorsebio.com/learning/app-notes/atp-vs-bioenergetic.php. Understanding the Relationship Between Bioenergetic Rates and ATP Turnover [Internet]. Seahorse Bioscience; 2013. Available from: http://www.seahorsebio.com/learning/app-notes/atp-vs-bioenergetic.php.

[21] Brown RP, Delp MD, Lindstedt SL, Rhomberg LR, Beliles RP (1997). Physiological parameter values for physiologically based pharmacokinetic models. Toxicol Ind Health.

[22] Perry SW, Norman JP, Barbieri J, Brown EB, Gelbard HA (2011). Mitochondrial membrane potential probes and the proton gradient: a practical usage guide. BioTechniques.

[23] Palmeira CM, Moreno AJ, Madeira VM, Wallace KB (1996). Continuous monitoring of mitochondrial membrane potential in hepatocyte cell suspensions. J Pharmacol Toxicol Methods.

[24] Berthiaume F, MacDonald AD, Kang YH, Yarmush ML (2003). Control analysis of mitochondrial metabolism in intact hepatocytes: effect of interleukin-1β and interleukin-6. Metab Eng.

[25] Leverve XM, Fontaine E, Putod-Paramelle F, Rigoulet M (1994). Decrease in cytosolic ATP/ADP ratio and activation of pyruvate kinase after *in vitro* addition of almitrine in hepatocytes isolated from fasted rats. Eur J Biochem FEBS.

[26] Abe Y, Sakairi T, Kajiyama H, Shrivastav S, Beeson C, Kopp JB (2010). Bioenergetic characterization of mouse podocytes. Am J Physiol Cell Physiol.

[27] Shoda LKM, Woodhead JL, Siler SQ, Watkins PB, Howell BA (2014). Linking physiology to toxicity using DILIsym(®), a mechanistic mathematical model of drug-induced liver injury. Biopharm Drug Dispos.

[28] Seitz HJ, Müller MJ, Krone W, Tarnowski W (1977). Coordinate control of intermediary metabolism in rat liver by the insulin/glucagon ratio during starvation and after glucose refeeding. Regulatory significance of long-chain acyl-CoA and cyclic AMP. Arch Biochem Biophys.

[29] Rossignol R, Gilkerson R, Aggeler R, Yamagata K, Remington SJ, Capaldi RA (2004). Energy substrate modulates mitochondrial structure and oxidative capacity in cancer cells. Cancer Res.

[30] Hue L, Sobrino F, Bosca L (1984). Difference in glucose sensitivity of liver glycolysis and glycogen synthesis. Relationship between lactate production and fructose 2,6-bisphosphate concentration. Biochem J.

[31] Johnson-Cadwell LI, Jekabsons MB, Wang A, Polster BM, Nicholls DG (2007). “Mild Uncoupling” does not decrease mitochondrial superoxide levels in cultured cerebellar granule neurons but decreases spare respiratory capacity and increases toxicity to glutamate and oxidative stress. J Neurochem.

[32] Hatafi Y. The structural basis of membrane function. Academic Press; 2012. 501 p.

[33] Schmitz JPJ, Groenendaal W, Wessels B, Wiseman RW, Hilbers PAJ, Nicolay K (2013). Combined in vivo and in silico investigations of activation of glycolysis in contracting skeletal muscle. Am J Physiol Cell Physiol.

[34] Nieminen AL, Saylor AK, Herman B, Lemasters JJ (1994). ATP depletion rather than mitochondrial depolarization mediates hepatocyte killing after metabolic inhibition. Am J Physiol.

[35] Weinberg F, Hamanaka R, Wheaton WW, Weinberg S, Joseph J, Lopez M (2010). Mitochondrial metabolism and ROS generation are essential for Kras-mediated tumorigenicity. Proc Natl Acad Sci.

[36] Gohil VM, Sheth SA, Nilsson R, Wojtovich AP, Lee JH, Perocchi F (2010). Nutrient-sensitized screening for drugs that shift energy metabolism from mitochondrial respiration to glycolysis. Nat Biotechnol.

[37] Beard DA. A Biophysical model of the mitochondrial respiratory system and oxidative phosphorylation. PLoS Comput Biol [Internet]. 2005 [cited 2013 May 5];1(4). Available from: http://www.ncbi.nlm.nih.gov/pmc/articles/PMC1201326/. 10.1371/journal.pcbi.0010036 PMC1201326 16163394

[38] Cortassa S, Aon MA, Marbán E, Winslow RL, O’Rourke B (2003). An integrated model of cardiac mitochondrial energy metabolism and calcium dynamics. Biophys J.

[39] Wei A-C, Aon MA, O’Rourke B, Winslow RL, Cortassa S (2011). Mitochondrial energetics, pH regulation, and ion dynamics: a computational-experimental approach. Biophys J.

[40] Dash RK, DiBella JA, Cabrera ME (2007). A computational model of skeletal muscle metabolism linking cellular adaptations induced by altered loading states to metabolic responses during exercise. Biomed Eng OnLine.

