# Supplementary material for: MITOsym®: A Mechanistic, Mathematical Model of Hepatocellular Respiration and Bioenergetics
Source: Pharm Res. 2014 Dec 12;32(6):1975–92. doi: 10.1007/s11095-014-1591-0 (PMC4422870; doi:10.1007/s11095-014-1591-0)
Supplement: Supplementary file 1 — (DOCX 37 kb) [file 11095_2014_1591_MOESM1_ESM.docx]

Appendix A

**Intermediate fluxes**

1. ${Flux}_{prod}^{lac}$ : ${Flux}_{prod}^{lac}$ is derived based on the ECAR values, plating conditions, and estimated cell volumes. Given the ECAR is $66.2\frac{\mathrm{mpH}}{\min}$, and conversion factor of $4.28 \frac{\mathrm{pmole}H^{+}}{\mathrm{mpH}}$ [1]. One can compute the basal ${Flux}_{prod}^{lac}$(mM/min) as:

${Flux}_{prod}^{lac}$ $=$66.2 $\frac{\mathrm{mpH}}{\min}\times4.28\frac{\mathrm{pmole}H^{+}}{\mathrm{mpH}}\div9E^{-8}\frac{L}{24000 cell}\div1E^{-9}\frac{\mathrm{mmole}}{\mathrm{pmole}}$~ 3.15 mM/min

1. ${Flux}_{prod, cellular}^{pyr}$*:* ${Flux}_{prod, cellular}^{pyr}$ is calculated bases the percentage of total glycolysis flux used for lactate production.

${Flux}_{prod}^{lac}$*=* ${Flux}_{prod, cellular}^{pyr}*(1-0.16$*),* therefore ${Flux}_{prod, cellular}^{pyr}$ *~* 3.75 mM/min

Note the fraction 0.16 is derived based on initial estimation of ATP contributed from two sources, OXPHOS and glycolysis. The ECAR-based cytosolic ATP production is approximately 283 pmole ATP/min (66.2 $\frac{\mathrm{mpH}}{\min}\times4.28\frac{\mathrm{pmole}H^{+}}{\mathrm{mpH}})$; and OXPHO ATP is approximately 1000 pmole ATP/min (199.6 $\left( \frac{\mathrm{pmoleO}_{2}}{\min} \right)\times5\left( \frac{\mathrm{pmoleATP}}{\mathrm{pmoleO}_{2}} \right)$[2]). Also, presumed glucose yield of 36 ATP during mitochondrial oxidation and 2 ATP during glycolysis [3], one could calculate the percentage (K) of total glycolysis flux used for mitochondrial oxidation as:

$\frac{2*\left( 1-K \right)}{2*\left( 1-K \right)+36*K} \sim\frac{283}{1281} \underset{\to}{yields} K\sim16\%$

1. ${Flux}_{prod, mito}^{pyr}: {Flux}_{prod, mito}^{pyr} is 0.6 mM/\min at basal state$*,* a difference between ${Flux}_{prod, cellular}^{pyr} and {Flux}_{prod}^{lac}$
2. ${Flux}_{prod}^{G6P}: {Flux}_{prod}^{G6P}$is 1.875 mM/min at basal state, since one G6P produces two pyruvate.
3. ${Flux}_{prod}^{pyr2OX}:$ ${Flux}_{prod}^{pyr2OX}$ is 0.6 mM/min at basal state, assuming rapid and complete flux between mitochondrial pyruvate uptake and electron transport chain flux.
4. ${Flux}_{prod}^{ETC}$: ${Flux}_{prod}^{ETC}$ is 0.6 mM/min at basal state, assuming rapid dynamics between mitochondrial oxidative phosphorylation and ETC activity. ETC activity is used as a surrogate marker for OCR measurement where OCR is computed as:

basal OCR*$\frac{{Flux}_{prod}^{ETC}}{{basal Flux}_{prod}^{ETC}}$ where basal OCR is 199.6 pmole O2/min/24000 cell

1. ${{Flux}_{prod, cellular}^{ATP} and Flux}_{prod,mito}^{ATP}$ $\mathrm{Flux}_{prod,mito}^{\mathrm{ATP}}$ at basal state is calculated as:

${Flux}_{prod,mito}^{ATP}$ =basal OCR $\left( \frac{\mathrm{pmoleO}_{2}}{\min} \right)\times5\left( \frac{\mathrm{pmoleATP}}{\mathrm{pmoleO}_{2}} \right)$~ 1000 pmole ATP/min

${Flux}_{prod, cellular}^{ATP}$= $\frac{{Flux}_{prod, cellular}^{pyr}}{{Flux}_{prod}^{lac}}\times66.2\left( \frac{\mathrm{mpH}}{\min} \right)\times4.28\left( \frac{\mathrm{pmole}H^{+}}{\mathrm{mpH}} \right)\times1(\frac{\mathrm{pmoleATP}}{\mathrm{pmole}H^{+}})$ ~336 pmole ATP/min

Define: ATP production (pmole/min) = ${Flux}_{prod,mito}^{ATP}$ + ${Flux}_{prod, cellular}^{ATP}$

The total ATP production rate is 1336 pmole/min at basal steady state.

References:

[1] http://www.seahorsebio.com/learning/app-notes/atp-vs-bioenergetic.php, “Understanding the Relationship Between Bioenergetic Rates and ATP Turnover.” Seahorse Bioscience, 2013.

[2] W. Zhou, M. Choi, D. Margineantu, L. Margaretha, J. Hesson, C. Cavanaugh, C. A. Blau, M. S. Horwitz, D. Hockenbery, C. Ware, and H. Ruohola-Baker, “HIF1α induced switch from bivalent to exclusively glycolytic metabolism during ESC-to-EpiSC/hESC transition,” *EMBO J.*, vol. 31, no. 9, pp. 2103–2116, May 2012.

[3] J. M. Berg, J. L. Tymoczko, and L. Stryer, *Biochemistry*, 5th ed. 2002.

[4] S. Nadanaciva, P. Rana, G. C. Beeson, D. Chen, D. a. Ferrick, C. C. Beeson, and Y. Will, “Assessment of drug-induced mitochondrial dysfunction via altered cellular respiration and acidification measured in a 96-well platform,” *J. Bioenerg. Biomembr.*, vol. 44, no. 4, pp. 421–437, Jun. 2012.

Appendix B- Sets of differential and algebraic equations used to describe glycolysis and mitochondrial ETC pathways (Corresponding with expressions listed in Table II-IV)

| $\frac{\boldsymbol{d}\left[ \boldsymbol{G}\boldsymbol{6}\boldsymbol{P} \right]}{\boldsymbol{dt}}$  **where** | $\boldsymbol{=}\boldsymbol{Eff\_Flux}_{\boldsymbol{prod}}^{\boldsymbol{G}\boldsymbol{6}\boldsymbol{P}}\boldsymbol{-}$ $\boldsymbol{Eff\_Flux}_{\boldsymbol{prod, cellular}}^{\boldsymbol{pyr}}\boldsymbol{*0.5-}$ $\boldsymbol{Flux}_{\boldsymbol{prod}}^{\boldsymbol{gly}}\boldsymbol{+}\boldsymbol{Flux}_{\boldsymbol{degrad}}^{\boldsymbol{gly}}$  $\boldsymbol{Eff\_Flux}_{\boldsymbol{prod}}^{\boldsymbol{G}\boldsymbol{6}\boldsymbol{P}}\boldsymbol{=}\frac{\boldsymbol{Vmax}_{\boldsymbol{g}\boldsymbol{6}\boldsymbol{p}}\boldsymbol{\times}\left[ \boldsymbol{Media} \right]}{\boldsymbol{Km}_{\boldsymbol{g}\boldsymbol{6}\boldsymbol{p}}\boldsymbol{+}\left[ \boldsymbol{Media} \right]}\boldsymbol{\times}\frac{{\mathbf{Km}_{\mathbf{f}\boldsymbol{b}_{\mathbf{G6P2Glu}}}}^{\boldsymbol{n}}\boldsymbol{+}{{\boldsymbol{[G}\boldsymbol{6}\boldsymbol{P}}_{\boldsymbol{basal}}\boldsymbol{]}}^{\boldsymbol{n}}}{{\mathbf{Km}_{\mathbf{f}\boldsymbol{b}_{\mathbf{G6P2Glu}}}}^{\boldsymbol{n}}\boldsymbol{+}{\boldsymbol{[G}\boldsymbol{6}\boldsymbol{P]}}^{\boldsymbol{n}}}$  $\boldsymbol{Eff\_Flux}_{\boldsymbol{prod, cellular}}^{\boldsymbol{pyr}}\boldsymbol{=}\frac{\boldsymbol{Vmax}_{\boldsymbol{pyu}}\boldsymbol{\times[G}\boldsymbol{6}\boldsymbol{P]}}{\boldsymbol{Km}_{\boldsymbol{pyu}}\boldsymbol{+[G}\boldsymbol{6}\boldsymbol{P]}}\boldsymbol{\times}\left( \left( \mathbf{1-K} \right)\boldsymbol{+K\cdot}\left( \frac{\boldsymbol{2\times}\mathbf{Basal}_{\mathbf{ATP}_{\mathbf{total}}}}{\mathbf{Basal}_{\mathbf{ATP}_{\mathbf{total}}}\mathbf{+}\left[ \boldsymbol{ATP} \right]_{\boldsymbol{total}}} \right) \right)\boldsymbol{\times}$  $\left( \boldsymbol{1+}\boldsymbol{K\cdot abs}\left( \frac{\boldsymbol{Flux}_{\boldsymbol{prod}}^{\boldsymbol{ETC}}}{{\boldsymbol{Flux}_{\boldsymbol{prod}}^{\boldsymbol{ETC}}}_{\boldsymbol{basal}}}\boldsymbol{-1} \right) \right)$  $\boldsymbol{Flux}_{\boldsymbol{prod}}^{\boldsymbol{gly}}\boldsymbol{=}\boldsymbol{K}_{\boldsymbol{prod}}^{\boldsymbol{gly}}\boldsymbol{\bullet[G}\boldsymbol{6}\boldsymbol{P]}$  $\boldsymbol{Flux}_{\boldsymbol{degrad}}^{\boldsymbol{gly}}\boldsymbol{=}\boldsymbol{K}_{\boldsymbol{degrad}}^{\boldsymbol{gly}}\boldsymbol{\bullet[Glycogen]}$ |
| --- | --- |
| $\frac{\boldsymbol{d}\boldsymbol{[Pyr}_{\boldsymbol{cellular}}\boldsymbol{]}}{\boldsymbol{dt}}$  **where** | $\boldsymbol{=}\boldsymbol{Eff\_Flux}_{\boldsymbol{prod, cellular}}^{\boldsymbol{pyr}}\boldsymbol{-}$ $\boldsymbol{Eff\_Flux}_{\boldsymbol{prod, mito}}^{\boldsymbol{pyr}}$ $\boldsymbol{-Flux}_{\boldsymbol{prod}}^{\boldsymbol{lac}}$  $\boldsymbol{Eff\_Flux}_{\boldsymbol{prod, mito}}^{\boldsymbol{pyr}}\boldsymbol{=}\boldsymbol{Eff\_Flux}_{\boldsymbol{prod, cellular}}^{\boldsymbol{pyr}}\boldsymbol{\times}\frac{{\mathbf{Km}_{\mathbf{f}\boldsymbol{b}_{\mathbf{mitoPyr}}}}^{\boldsymbol{n}}\boldsymbol{+}{\boldsymbol{[Pyu}_{\boldsymbol{mito}}^{\boldsymbol{basal}}\boldsymbol{]}}^{\boldsymbol{n}}}{{\mathbf{Km}_{\mathbf{f}\boldsymbol{b}_{\mathbf{mito}\mathbf{Pyr}}}}^{\boldsymbol{n}}\boldsymbol{+}{\boldsymbol{[Pyu}_{\boldsymbol{mito}}\boldsymbol{]}}^{\boldsymbol{n}}}$  $\boldsymbol{Flux}_{\boldsymbol{prod}}^{\boldsymbol{lac}}\boldsymbol{=}\boldsymbol{K}_{\boldsymbol{lac}}\boldsymbol{\bullet}\boldsymbol{[Pyr}_{\boldsymbol{cellular}}\boldsymbol{]}$ |
| $\frac{\boldsymbol{d}\boldsymbol{[Pyr]}_{\boldsymbol{mito}}}{\boldsymbol{dt}}$  **where** | $\boldsymbol{=Eff\_Flux}_{\boldsymbol{prod, mito}}^{\boldsymbol{pyr}}\boldsymbol{-}$ $\boldsymbol{Eff\_Flux}_{\boldsymbol{prod}}^{\boldsymbol{pyr}\boldsymbol{2}\boldsymbol{OX}}$  $\boldsymbol{Eff\_Flux}_{\boldsymbol{prod}}^{\boldsymbol{pyr}\boldsymbol{2}\boldsymbol{OX}}\boldsymbol{=}\frac{\boldsymbol{Vmax}_{\boldsymbol{mitoOX}}\boldsymbol{\times}\boldsymbol{[Pyr]}_{\boldsymbol{mito}}}{\boldsymbol{Km}_{\boldsymbol{mitoOX}}\boldsymbol{+}\boldsymbol{[Pyr]}_{\boldsymbol{mito}}}\boldsymbol{\times}\frac{{\mathbf{Km}_{\mathbf{f}\boldsymbol{b}_{\mathbf{mitoOX}}}}^{\boldsymbol{n}}\boldsymbol{+}{\boldsymbol{[OX}_{\boldsymbol{mito}}^{\boldsymbol{basal}}\boldsymbol{]}}^{\boldsymbol{n}}}{{\mathbf{Km}_{\mathbf{f}\boldsymbol{b}_{\mathbf{mitoOX}}}}^{\boldsymbol{n}}\boldsymbol{+}{\boldsymbol{[OX}_{\boldsymbol{mito}}\boldsymbol{]}}^{\boldsymbol{n}}}\boldsymbol{\times}$ $\frac{\boldsymbol{Km\_NegFeedA}^{\boldsymbol{n}}}{\boldsymbol{Km\_NegFeedA}^{\boldsymbol{n}}\boldsymbol{+}\mathbf{m}^{\boldsymbol{n}}}$ |
| $\frac{\boldsymbol{d}\left[ \boldsymbol{OX} \right]_{\boldsymbol{mito}}}{\boldsymbol{dt}}$  **where** | $\boldsymbol{=Eff\_Flux}_{\boldsymbol{prod}}^{\boldsymbol{pyr}\boldsymbol{2}\boldsymbol{OX}}\boldsymbol{-}\boldsymbol{Eff\_Flux}_{\boldsymbol{prod}}^{\boldsymbol{ETC}}$  $\boldsymbol{Eff\_Flux}_{\boldsymbol{prod}}^{\boldsymbol{ETC}}\boldsymbol{=}\boldsymbol{K}_{\boldsymbol{ETC}}\boldsymbol{\bullet}\boldsymbol{[OX}_{\boldsymbol{mito}}\boldsymbol{]\bullet} \mathbf{Drug}_{\boldsymbol{ETC\_inhibitor}}$  $\mathbf{Drug}_{\boldsymbol{ETC\_inhibitor}}\boldsymbol{=}\frac{\boldsymbol{Ki}_{\boldsymbol{ETC\_inhibitor}}}{\boldsymbol{Ki}_{\boldsymbol{ETC\_inhibitor}}\boldsymbol{+}\boldsymbol{[Drug]}_{\boldsymbol{ETC\_inhibitor}}}$ |
| $\frac{\boldsymbol{d}\boldsymbol{\Psi}_{\boldsymbol{m}}}{\boldsymbol{dt}}$  **where** | $\boldsymbol{=Coversion}_{\boldsymbol{f}}\boldsymbol{\times Eff\_}\boldsymbol{Flux}_{\boldsymbol{prod}}^{\boldsymbol{ETC}}\boldsymbol{-}$ $\boldsymbol{Eff\_Flux}_{\boldsymbol{prod,mito}}^{\boldsymbol{ATP}}\boldsymbol{-Uncoupling}_{\boldsymbol{drug}}$  $\boldsymbol{Eff\_Flux}_{\boldsymbol{prod,mito}}^{\boldsymbol{ATP}}\boldsymbol{=}\frac{\boldsymbol{Vmax}_{\boldsymbol{ATP}}\boldsymbol{\times}\boldsymbol{\Psi}_{\boldsymbol{m}}}{\boldsymbol{Km}_{\boldsymbol{ATP}}\boldsymbol{+}\boldsymbol{\Psi}_{\boldsymbol{m}}}\boldsymbol{\times}\frac{\boldsymbol{2\bullet BasalFlux}_{\mathbf{ATP}_{\mathbf{total}}}}{\mathbf{BasalFlux}_{\mathbf{ATP}_{\mathbf{total}}}\mathbf{+}\mathbf{CurrentFlux}_{\mathbf{ATP}_{\mathbf{total}}}}$ $\boldsymbol{\times}\mathbf{Drug}_{\boldsymbol{ATPase\_inhibitor}}$  $\mathbf{Drug}_{\boldsymbol{ATPase\_inhibitor}}\boldsymbol{=}\frac{\boldsymbol{Ki}_{\boldsymbol{ATPase\_inhibitor}}}{\boldsymbol{Ki}_{\boldsymbol{ATPase\_inhibitor}}\boldsymbol{+}\boldsymbol{[Drug]}_{\boldsymbol{ETC\_inhibitor}}}{ \atop}$  $\boldsymbol{Uncoupling}_{\boldsymbol{drug}}\boldsymbol{=}\frac{\boldsymbol{Vmax}_{\boldsymbol{UC}}\boldsymbol{\times}\boldsymbol{[Drug]}_{\boldsymbol{uncoupler}}}{\boldsymbol{Km}_{\boldsymbol{UC}}\boldsymbol{+}\boldsymbol{[Drug]}_{\boldsymbol{uncoupler}}}\boldsymbol{\times}$ $\boldsymbol{\Psi}_{\boldsymbol{m}}$ |
| $\frac{\boldsymbol{d}\left[ \boldsymbol{ATP} \right]_{\boldsymbol{cellular}}}{\boldsymbol{dt}}$  **where** | $\boldsymbol{=Eff\_Flux}_{\boldsymbol{mito, lowGrad}}^{\boldsymbol{ATP}}\boldsymbol{+}$ $\boldsymbol{Eff\_Flux}_{\boldsymbol{gly}}^{\boldsymbol{ATP}}\boldsymbol{-}\boldsymbol{k*}\boldsymbol{[ATP]}_{\boldsymbol{cellular}}$  ${\boldsymbol{Ef}\boldsymbol{f}_{\boldsymbol{Flux}}}_{\boldsymbol{mito, lowGrad}}^{\boldsymbol{ATP}}\boldsymbol{=}{\boldsymbol{Ef}\boldsymbol{f}_{\boldsymbol{Flux}}}_{\boldsymbol{prod,mito}}^{\boldsymbol{ATP}}$ $\boldsymbol{\times}\left( \boldsymbol{1-k\bullet}\left( \frac{\left( \mathbf{Km}_{\mathbf{MMP2ATP}} \right)^{\mathbf{n}}}{\left( {\mathbf{Km}_{\mathbf{MMP2ATP}}}^{\mathbf{n}}\mathbf{+}\left( \frac{\mathbf{m}_{\mathbf{basal}}}{\mathbf{m}} \right)^{\mathbf{n}} \right)}\mathbf{-}\frac{\left( \mathbf{Km}_{\mathbf{MMP2ATP}} \right)^{\mathbf{n}}}{{\mathbf{(Km}_{\mathbf{MMP2ATP}}}^{\mathbf{n}}\mathbf{+1)}} \right) \right)$  $\boldsymbol{Eff\_Flux}_{\boldsymbol{gly}}^{\boldsymbol{ATP}}\boldsymbol{=}\boldsymbol{Eff\_Flux}_{\boldsymbol{prod, cellular}}^{\boldsymbol{pyr}}\boldsymbol{\times}\left( \boldsymbol{1-K\cdot}\left( \frac{{\boldsymbol{S}_{\boldsymbol{f}\boldsymbol{b}_{\boldsymbol{ATP}\boldsymbol{2}\boldsymbol{gly}}}}_{\boldsymbol{current}}}{{\boldsymbol{S}_{\boldsymbol{f}\boldsymbol{b}_{\boldsymbol{ATP}\boldsymbol{2}\boldsymbol{gly}}}}_{\boldsymbol{basal}}}\boldsymbol{\times}\frac{{\boldsymbol{S}_{\boldsymbol{f}\boldsymbol{b}_{\boldsymbol{ETC}\boldsymbol{2}\boldsymbol{gly}}}}_{\boldsymbol{current}}}{{\boldsymbol{S}_{\boldsymbol{f}\boldsymbol{b}_{\boldsymbol{ETC}\boldsymbol{2}\boldsymbol{gly}}}}_{\boldsymbol{basal}}}\boldsymbol{-1} \right) \right)$ |
|  |  |
|  |  |
|  |  |

Appendix C

**Supplement Table.** The fold of change in the measured oxygen consumption rate (OCR), extracellular acidification rate (ECAR) and mitochondria membrane potential of HepG2 cells on addition of rotenone, FCCP and oligomycin. For OCR and ECAR, the data were collected using XF96 analyzer after HepG2 were incubated for 15-60 min at various concentrations of rotenone, FCCP and oligomycin. For membrane potential, the TMRM accumulation was measured after incubation in fresh medium containing FCCP at various concentrations for 1 hour. All cells were cultured with 5.5 mM glucose, 2 mM glutamine and 10% fetal bovine serum.

| Table S1 | Rotenone | | |  |
| --- | --- | --- | --- | --- |
|  | uM | OCR^†^ | ECAR^†^ |  |
|  | 0.0005 | 0.9969 | 1.0 |  |
|  | 0.015 | 0.9514 | 1.0 |  |
|  | 0.03 | 0.8739 | 1.1 |  |
|  | 0.06 | 0.8151 | 1.2 |  |
|  | 0.12 | 0.7082 | 1.3 |  |
|  | 0.25 | 0.4998 | 1.5 |  |
|  | 0.5 | 0.318 | 1.5 |  |
|  | 1 | 0.2 | 1.6 |  |

†Data previously shown in Nadanaciva et al J Bioenerg Biomembr 2012

| Table S2 | FCPP | | | | |  |
| --- | --- | --- | --- | --- | --- | --- |
|  | uM | OCR^†^ | ECAR^†^ | uM | MMP^‡^ |  |
|  | 0.0005 | 100 | 1 | 0.005 | 0.999 |  |
|  | 0.05 | 100 | 1.2 | 0.012 | 0.931 |  |
|  | 0.141 | 140 | 1.39 | 0.037 | 0.921 |  |
|  | 0.25 | 220 | 2 | 0.111 | 1.05 |  |
|  | 0.5 | 390 | 2.6 | 0.333 | 1.14 |  |
|  | 1 | 400 | 2.5 | 1 | 0.949 |  |
|  |  |  |  | 3 | 0.621 |  |
|  |  |  |  | 9 | 0.276 |  |

†Data previously shown in Nadanaciva et al J Bioenerg Biomembr 2012

‡Experimental measured data, see Method section

| Table S3 | Oligomycin | | |  |
| --- | --- | --- | --- | --- |
|  |  | OCR^‡^ | ECAR^‡^ |  |
|  | 0.005 | 0.97 | 1.0 |  |
|  | 0.125 | 0.987 | 1.1 |  |
|  | 0.25 | 0.982 | 1.1 |  |
|  | 0.5 | 0.963 | 1.1 |  |
|  | 1 | 0.517 | 1.4 |  |
|  | 2 | 0.264 | 1.7 |  |

‡Experimental measured data, see Method section
